# Noncanonical function of epigenetic reader YTHDF1 inhibits MASLD progression by maintaining peroxisomes and mitochondrial homeostasis

**DOI:** 10.1038/s12276-026-01686-3

**Published:** 2026-04-10

**Authors:** Chenyang Mu, Jian Tan, Yuefan Wang, Haozan Yin, Zhihui Dai, Zenghan Wang, Dongyang Ding, Zhichao Zhang, Sijie Wang, Hui Liu, Fu Yang

**Affiliations:** 1 Department of Medical Genetics, Naval Medical University, Shanghai, China; 2 School of Health Science and Engineering, University of Shanghai for Science and Technology, Shanghai, China; 3 Third Department of Hepatic Surgery, Eastern Hepatobiliary Surgery Hospital, Naval Medical University, Shanghai, China; 4 Research Institute of General Surgery, Nanjing University School of Medicine, General Hospital of Eastern Theater Command PLA, Nanjing, China; 5 Shanghai Key Laboratory of Medical Bioprotection, Shanghai, China; 6 Key Laboratory of Biological Defense, Ministry of Education, Shanghai, China

**Keywords:** Non-alcoholic fatty liver disease, Experimental models of disease

## Abstract

YTH *N*^6^-methyladenosine RNA binding protein F1 (YTHDF1) enables *N*^6^-methyladenosine-containing RNA binding activity, involved in mRNA destabilization and positive regulation of translational initiation. Here we aimed to investigate the main molecular events associated with YTHDF1 during the course of metabolic dysfunction-associated steatotic liver disease (MASLD). YTHDF1 expression was detected by western blotting and real-time PCR. *Ythdf1* hepatocyte-specific knockout mice were generated in this study. RNA sequencing and proteomic analyses were performed to investigate potentially involved molecular pathways. The protein levels of YTHDF1 were increased during MASLD progression. Under high-fat diet intervention, hepatocyte-specific *Ythdf1*-knockout mice exhibited a pronounced increase in both liver weight and liver-to-body weight ratio, accompanied by significant hepatic steatosis. YTHDF1 depletion promotes the progression of MASLD through enhanced peroxisome activation and mitochondria dysfunction, which are independent of its RNA *N*^6^-methyladenosine reader activity. In particular, decreased YTHDF1 enhances the expression of acyl-CoA oxidase 1 (ACOX1), and peroxisome activation in a manner relies on YTHDF1 facilitating the formation of stress granules in MASLD. In addition, YTHDF1 was localized in the mitochondria and interacted with SLC25A11, affecting mitochondrial glutathione transport and its homeostasis. Finally, the identified lysine 191 methylation modification can reduce the stability of YTHDF1 protein, thereby achieving its protein expression regulation during MASLD progression. YTHDF1 inhibits MASLD progression by modulating stress granule sequestration of *ACOX1* mRNA and maintaining mitochondrial homeostasis.

## YTHDF1 regulates mitochondrial homeostasis in liver disease

Metabolic dysfunction-associated steatotic liver disease (MASLD) is a common liver condition linked to obesity and lifestyle, often leading to liver cancer. Researchers are trying to understand MASLD better because there are no effective treatments yet. Researchers studied a protein called YTHDF1, which might help to manage MASLD. They used human liver samples and mice to explore how YTHDF1 affects the disease. The study found that YTHDF1 helps to control stress granules and maintains healthy mitochondria. In mice lacking YTHDF1, MASLD worsened owing to increased peroxisome activity and mitochondrial dysfunction. The researchers concluded that YTHDF1 plays a protective role in MASLD by regulating these cellular processes. This discovery could lead to new treatments targeting YTHDF1. Future research may explore how YTHDF1 can be used in therapies for liver diseases such as MASLD.

This summary was initially drafted using artificial intelligence, then revised and fact-checked by the author.

## Introduction

Metabolic dysfunction-associated steatotic liver disease (MASLD), which is directly related to metabolic dysfunction, has become one of the most common basic illnesses of liver cancer because of the prevalence of obesity and lifestyle^1–3^. MASLD is viewed as a range of liver illnesses, including liver steatosis, metabolic dysfunction-associated steatohepatitis (MASH) and cirrhosis^4,5^. In individuals with MASLD, the incidence of hepatocellular carcinoma was 0.44 per 1000 person-years, whereas it was 5.29 per 1000 person-years in patients with MASH according to a recent study^6^. Owing to a lack of understanding of the pathogenic mechanisms underlying MASLD, no effective pharmacotherapies are currently available, making MASLD a global health concern with enormous socioeconomic cost^7,8^.

Owing to increased fatty acid absorption and de novo lipogenesis in MASLD, compensatory oxidation augmentation is insufficient to restore normal lipid levels, generating oxidative stress characterized by impaired mitochondrial function and increased oxidation in peroxisomes^9,10^. Mitochondrial abnormalities have been repeatedly linked to the development and progression of liver steatosis and MASLD pathogenesis, but it is difficult to determine with certainty whether mitochondrial dysfunction or oxidative stress are the primary events or simply a byproduct of MASLD development^11^. In addition, peroxisomes, which catalyze the desaturation of acyl-CoA to 2-trans-enoyl-CoA via acyl-CoA oxidase 1 (ACOX1), play an essential role in MASLD^12^. Peroxisome-derived acetyl-CoA activated mTORC1, inhibiting lipophagy and thereby increasing hepatic steatosis^13^. Furthermore, peroxisome-derived reactive oxygen species (ROS) promote MASLD by increasing adipose triglyceride lipase degradation and decreasing lipolysis^14^. Oxidative stress resulting from elevated fatty acid oxidation leads to hepatic cell death and inflammation, while simultaneously activating stress granules (SGs) that may impede the advancement of MASLD^15^. SGs are formed from stalled mRNAs and contain a range of translation initiation factors, RNA-binding proteins and non-RNA-binding proteins^16^. Through the formation of SGs, nonessential transcripts can be sequestered and temporarily silenced in response to stress conditions, thereby affecting mRNA localization, translation and degradation^17^. Despite substantial research on oxidative stress in MASLD, the current comprehension of SGs caused by oxidative stress remains limited.

YTHDF1, a classic RNA *N*^6^-methyladenosine (m^6^A) reader, plays crucial roles in regulating cell stemness^18,19^, reprogramming tumor cell metabolism^20^ and tissue immune homeostasis^21^. Previous studies have shown that YTHDF1 promotes mRNA translation or RNA degradation in an m^6^A-dependent manner^22,23^. In our previous study, we reported that YTHDF1 is involved in the regulation of m^6^A-modified glutaminase 2 (GLS2) expression mediated by METTL14 in MASLD^24^. In recent years, the focus of research on YTHDF1 has shifted from its m^6^A recognition to exploring the regulatory factors that interact with YTHDF1 upstream and downstream to evaluate its potential as an intervention target^25^. Here, we show that YTHDF1 inhibits the progression of MASLD by modulating SG sequestration of *ACOX1* mRNA and maintaining mitochondrial homeostasis by positioning it in the mitochondria.

## Materials and methods

### Human samples

All of the MASLD and control liver tissues were collected as paratumor tissues from patients during hemangioma surgery. These patients were proven to have no history of liver illness from other causes, such as viral hepatitis or alcohol or drug use. The Ethics Committee of Eastern Hepatobiliary Surgical Hospital (EHBH) granted all participants’ written informed consent for sample collection (EHBHKY2016-01-018).

### Mice

All of the mice used in this study were raised in a pathogen-free setting at the Animal Center of Naval Medical University, which offered full access to food and water as well as a climate-controlled environment (22–25°C, 40–60% humidity). The Institutional Animal Care and Use Committee of Naval Medical University (Shanghai, China) gave its approval for all animal experiments to be carried out. Each experimental group included a minimum of three mice.

Conditional *Ythdf1*-knockout (KO) allele was produced by Cyagen Biosciences (www.cyagen.com). By combining albumin-Cre transgenic mice with *Ythdf1*^lox/lox^ mice, *Ythdf1* liver-specific KO mice were produced. Using the indicated primer sets, mice were genotyped.

Mice at 8 weeks of age were fed three different diets, respectively, to create a high-fat diet (HFD)-fed, Western diet (WD)-fed and amylin liver nonalcoholic steatohepatitis-inducing diet (AMLN)-fed MASLD mouse model: the HFD, which contained 60 kcal% fat, 20 kcal% protein and 20 kcal% carbohydrate (HF60, Dyets); the WD, which had 41% sucrose and 1.25% cholesterol and included 40 kcal% fat, 17 kcal% protein and 43 kcal% carbohydrate (D18061501, Dyets); and the AMLN, which had 40 kcal% fat, 22% fructose and 2% cholesterol (AMLN, Dyets). The standard chow diet served as the control. The weight of the mice was recorded weekly. The mice were killed 16 weeks later, and the liver tissues were saved for further research.

### Cell lines

Cells were grown in DMEM or MEM media (Gibco) supplemented with 10% fetal bovine serum (Gibco), 1% penicillin, streptomycin (Invitrogen) and antiprophylactic treatment (Invivogen) at 37 °C in a 5% CO_2_ humid environment. To induce steatosis in cells, sodium palmitate and sodium oleate were used.

### Lentiviral infection and plasmid transfection

Overexpressed YTHDF1 lentivirus named PGMLV-CMV-H_YTHDF1-3×Flag-EF1-ZsGreen1-T2A-Puro, interfering lentivirus named pLKD-CMV-EGFP-2A-Puro-U6-shRNA (YTHDF1), plasmids of overexpressed YTHDF1 named pSLenti-EF1-EGFP-P2A-Puro-CMV-YTHDF1-3xFLAG-WPRE, corresponding mutation types inserting the sequencing of K395A, Y397A named pSLenti-EF1-EGFP-P2A-Puro-CMV-YTHDF1(K395A, Y397A)-3xFLAG-WPRE and mutation types inserting the sequencing of K191R named pSLenti-EF1-EGFP-P2A-Puro-CMV-YTHDF1(K191R)-3xFLAG-WPRE were constructed by Obio Biosciences (www.obiosh.com). Lentivirus vectors were directly added to the cells inoculated in six-well plates for lentiviral infection, while plasmid transfection required the use of lipofectamine 3000 (Invitrogen). After 48 h of cell culture, the medium was replaced and the effectiveness of the transfection was assessed. Lentivirus-infected cells required 2 weeks of 2 μg ml^−1^ puromycin treatments.

### Western blotting

Hepatic tissues and cells were lysed in RIPA buffer (Beyotime) to extract proteins. Total proteins were denatured by boiling them in loading buffer. Protein samples were then separated by sodium dodecyl sulfate–polyacrylamide gel electrophoresis (SDS–PAGE) and transferred to nitrocellulose membranes. After blocking the membranes with fast block solution (Epizyme), they were treated with primary antibodies. Finally, the membranes were treated with fluorescent secondary antibodies before being scanned with an Odyssey infrared fluorescence scanner (LICOR). The details of antibodies are presented in Supplementary Table 3.

### qRT–PCR

RNA isolation kits (Vazyme, #R701) or TRIzol reagents (Thermo Fisher) were used to isolate total RNA from cells and tissues. Following concentration determination, total RNA was reverse transcribed into cDNA with reverse transcription reagents (Takara, #RR036A). Then, using ChamQ Universal SYBR qPCR Master Mix (Vazyme, #Q771-02), quantitative real-time PCR (qRT–PCR) was performed. Using the 2^−δδ^ct approach, relative gene expression was standardized to the expression of 18S or β-actin. The sequence of qRT–PCR primers is presented in Supplementary Table 4.

### IHC

Immunohistochemistry (IHC) assays were performed on mouse hepatic tissues that had been fixed in formaldehyde and embedded in paraffin. Before blocking with 10% goat serum, the implanted tissues were dewaxed to recover antigens. They were then treated with primary antibodies, followed by secondary antibodies; the antibody information is presented in Supplementary Table 3. Finally, nuclei were dyed with 3,3′-diaminobenzidine treatment and hematoxylin staining. Image J was used to quantify the expression levels of the targeted proteins after the sections were scanned and assessed.

### Immunofluorescence

The cells were rinsed in PBS before being fixed in 4% paraformaldehyde. Following permeabilization with 0.25% Triton X-100, cells were blocked via quick block solution (Epizyme) and treated with primary antibodies overnight. The cells were then rinsed in PBS before being treated with secondary antibodies. Finally, the nuclei of the cells were labeled with DAPI and the images were snapped with a Zeiss LSM900 confocal microscope.

### RNA FISH

After the cells were fixed with 4% paraformaldehyde, RNA fluorescent in situ hybridization (FISH) was performed using the Ribo Fluorescent In Situ Hybridization Kit (RIOBIO, R11060.7), and all of the following procedures were carried out according to the manufacturer’s protocol. In brief, cells were penetrated before being treated with FISH probe mix. Finally, the cell nuclei were stained with DAPI and images were captured using a Zeiss LSM900 confocal microscope.

### Protein IP

Immunoprecipitation Kit with Protein A+G Magnetic Beads (Beyotime, P2179S) was used for protein immunoprecipitation (IP). To summarize, cells were collected and resuspended in lysis buffer to produce lysate, and 50 μl of lysate was saved as input. Protein A+G Magnetic Beads were subjected to incubation overnight with antibodies before being treated with lysate. The proteins were then eluted using SDS–PAGE sample loading buffer, and the eluted samples were stored as IP. Finally, western blotting was used for analyzing IP and input.

### RIP

Using the Magna RIP RNA-Binding Protein Immunoprecipitation Kit (17-700, Merck), RNA IP was performed. All of the procedures were carried out exactly as instructed. Cells were collected and suspended in RNA-binding protein IP (RIP) lysis buffer before centrifugation to get supernatant. The Magnetic Beads Protein A/G were then treated with antibodies before being mixed with the cell lysate supernatant. Finally, RNA was extracted from the beads for qRT–PCR analysis.

### ROS assay

Reactive Oxygen Species Assay Kit (CA1420, Solarbio) was used to perform the ROS assay. PBS was used to clean the cells before dihydroethidium treatment for an hour. Cells were rinsed with PBS once more after discarding the dihydroethidium, and then flow cytometry (CyAn ADP Analyzer, Beckman Coulter) and fluorescent microscopy (Olympus, #BX53) were used to detect them.

### Metabolite measurements

Metabolite assays were performed on hepatic tissues and plasma. Triglyceride (TG; BC0625, Solarbio), nonesterified fatty acid (BC0595, Solarbio), total cholesterol (TC; BC1985, Solarbio) and serum alanine aminotransferase (ALT; BC1555, Solarbio) were measured according to the manufacturer’s instructions.

### Oil Red O and hematoxylin and eosin staining

Modified Oil Red O Staining Kit (Beyotime, C0158S) was used for Oil Red O Staining. Fixed frozen tissue samples were washed in 60% isopropanol after being fixed in 4% paraformaldehyde. They were then cleaned with PBS and treated with Oil Red O Staining solution. They were subsequently stained with hematoxylin and photographed under a microscope. Paraffin sections were dewaxed and stained with hematoxylin. They were washed, stained with eosin and then dehydrated to seal. Then, they were photographed using a microscope.

### RNA-seq

Using the TRIzol reagent from Thermo Fisher, total RNA was isolated from mouse hepatic tissues. Oebiotech (www.Oebiotech.com) carried out the RNA sequencing (RNA-seq) and bioinformatic data analysis. For each gene, the expression value fragments per kilobase of exon model per million mapped fragments (FPKM) was determined by HTSeq. The differentially expressed genes were filtered using DESeq2 with the following criteria: fold change >2 and *q* < 0.05.

### SILAC

The coding sequence of the *YTHDF1* gene (NM_017798) was inserted into p3×FLAG-CMV-7.1 vector (Sigma-Aldrich) between NotI and BglII restriction sites (p3×FLAG-YTHDF1).

We maintained 293T cells in DMEM medium containing 13C6-lysine and 13C6-arginine (K6R6) supplemented with 10% dialyzed fetal bovine serum (FBS; Gibco) over eight passages to label the whole cellular proteome with stable isotope labeling with amino acids in cell culture (SILAC) approach (PMID: 12118079), and transfected with p3×FLAG-YTHDF1 plasmid overexpression cell (OE cell). By contrast, empty vector (p3×FLAG-CMV-7.1) was transfected into 293T cells grown in normal DMEM medium supplemented with 10% dialyzed FBS (Gibco) Negative control cell (NC cell). The transfected NC and OE cells were lysed with lysis buffer (20 mM Tris (pH 7.5), 150 mM NaCl, 1% Triton X-100) supplemented with protease and phosphatase inhibitor cocktail (Sigma-Aldrich) and protein yield was quantified using BCA assay kit (ThermoFisher Scientific). Equal amounts of proteins were incubated with anti-Flag beads (Sigma-Aldrich) to immunoprecipitate the protein interaction complex. The enriched protein interaction complexes from NC and OE cells were eluted by 3 × Flag peptide (Sigma-Aldrich) and combined, separated by SDS–PAGE gel, stained with Coomassie brilliant blue and cut into several bands of the whole lane for downstream in-gel trypsin digestion.

### Protein in-gel trypsin digestion

Following the protocol described previously (PMID: 17406544), the gel bands were cut into pieces and washed with Milli-Q water. Coomassie Brilliant Blue staining dye was removed with 50% acetonitrile (ACN)/50 mM ammonium bicarbonate. The gel slices were dehydrated twice in 100% ACN for 30 min and reconstituted in 50 mM ammonium bicarbonate containing 10 ng μl^−1^ sequencing-grade trypsin (Promega) to digest proteins at 37 °C for 16 h. The tryptic peptides were extracted from the gel pieces with 50% ACN/0.1% TFA and lyophilized by vacuum centrifugation. The dried peptides were resuspended in 0.1% formic acid and water solution and desalted using Monospin C18 column (GL Sciences).

### Liquid chromatography–MS/MS analysis

Liquid chromatography–tandem mass spectrometry analysis of peptide samples was performed on a nano-HPLC chromatography system (NanoElute, Bruker Daltonics) connected to a hybrid trapped ion mobility spectrometry quadrupole time-of-flight mass spectrometer (TIMS-TOF Pro, Bruker Daltonics) via a CaptiveSpray nano-electrospray ion source. A total of 200 ng peptides dissolved in solvent A (0.1% formic acid) was loaded onto the analytical column (75 µm inner diameter × 25 cm) and separated with a 60 min gradient (2–22% solvent B (ACN with 0.1% formic acid) for 45 min, 22–37% B for 5 min, 37−80% B for 5 min and then 80% B for 5 min). The flow rate was maintained at 300 nl min^−1^. For mass spectrometry (MS) analysis, the accumulation and ramp time were set as 100 ms each. Survey full-scan MS spectra (*m*/*z* 100–1700) were obtained in positive electrospray mode. The ion mobility was scanned from 0.7 to 1.3 Vs cm^−^^2^. The overall acquisition cycle of 1.16 s comprised one full trapped ion mobility spectrometry–MS scan and ten parallel accumulation–serial fragmentation (PASEF) tandem mass spectrometry (MS/MS) scans. During PASEF MS/MS scanning, the collision energy was ramped linearly as a function of the mobility from 59 eV at 1/*K*_0_ = 1.6 Vs cm^−^^2^ to 20 eV at 1/*K*_0_ = 0.6 Vs cm^−^^2^.

The MS raw file was processed using Fragpipe (v1.8) for protein identification and quantification (PMID: 28394336). Data were searched against the Human UniProtKB database (20,383 entries, release 2022_03). Statistical analysis of determination of YTHDF1-specific interactors was assessed using significance B (*P* ≥ 0.05), as defined using Perseus (version 2.0.3.1, http://www.perseusframework.org/) on the log_2_ H/L ratio (H: heavy-labeled condition; L: light-labeled condition).

### Mitochondrial colocalization imaging assay

Cells with a density of 1 × 10^5^ cells ml^–1^ were cultured in confocal cuvettes at 37 °C for 12 h. After the culture medium was removed, the cells were stained with MitoTracker Red probe (100 nM) for 20 min. Then, the cells were fixed using 4% paraformaldehyde various, and then designated antibodies (Supplementary Table 3) in an FBS-free cell culture medium were subsequently added into each chamber in sequence. After being further incubated with DAPI (10 μg ml^−1^) for 30 min, fluorescent images were captured by BioTek Cytation C10 (CC10, Agilent). DAPI was used with an excitation wavelength of 408 nm and an emission wavelength of 460 nm, while MitoTracker was used with an excitation wavelength of 579 nm and an emission wavelength of 610 nm.

### Mitochondrial membrane potential depolarization

Hep 3B cells with a density of 3 × 10^5^ cells/ml were cultured in confocal cuvettes under 5% CO_2_ at 37 °C. After 12 h, the cells were stained with 1 ml JC-1 probe (10 µg ml^−1^) for 30 min in dark. After washing with PBS for five times, the cells were then imaged by BioTek Cytation C10 (CC10, Agilent) to examine the fluorescence intensity of the J-monomers and the J-aggregates.

### OCR measurement

The oxygen consumption rate (OCR) was measured using a Seahorse instrument (Agilent). In brief, the sensor cartridge was hydrated a day before the test, and the cells were inoculated into a specific culture plate for overnight culture. Then, the cells were changed into the Seahorse detection liquid and incubated at 37 °C for 60 min without CO_2_ supply. The OCR was measured under basal conditions and with the addition of oligomycin (1 μM, introduced after 28 min), carbonyl cyanide *p*-trifluoromethoxy phenylhydrazone (FCCP, 0.5 μM, introduced after 44 min) and rotenone/antimycin A (Rot/AA, 0.5 μM, introduced after 80 min). Finally, the results of the OCR were obtained by data normalization processing. The base respiration was equal to the difference between the initial and termination values of the OCR, and the spare respiratory capacity was equal to the difference between the maximum and initial values of the OCR.

### Mitochondrial Bio-TEM morphology observation

A total of 1 × 10^6^ cells were plated in 10-cm dishes and then cultured in an incubator for 24 h. Then, the collected pellets were sequentially fixed with 2.5% glutaraldehyde and 1% OsO_4_ at 4 °C. Subsequently, the fixed samples were dehydrated sequentially using solutions of ethyl alcohol (50%, 75%, 90% and 100%) and acetone (100%) before embedding over a period of 24 h with increased concentrations of a resin in acetone (25%, 50%, 75% and 100%). Following this, cell slices of different groups were obtained in an ultramicrotome after a series of programmed treatments, which were then subsequently mounted onto copper grids, followed by staining with 2% uranyl acetate and lead citrate solution. The mitochondrial morphology in the different samples was observed via TEM.

### Statistical analysis

GraphPad Prism 9 software was applied to perform statistical analysis. The mean ± standard error of mean (s.e.m.) was used to present all the data for statistical analysis. To compare group differences, paired tests and unpaired *t*-tests were used. A *P* value below 0.05 was considered statistically significant. At least three independent repeats of each experiment were conducted. ^*^P < 0.05, ^**^P < 0.01, ^***^P < 0.001, ^****^P < 0.0001.

## Results

### The protein level of hepatic YTHDF1 is increased in MASLD

**Fig. 1:**
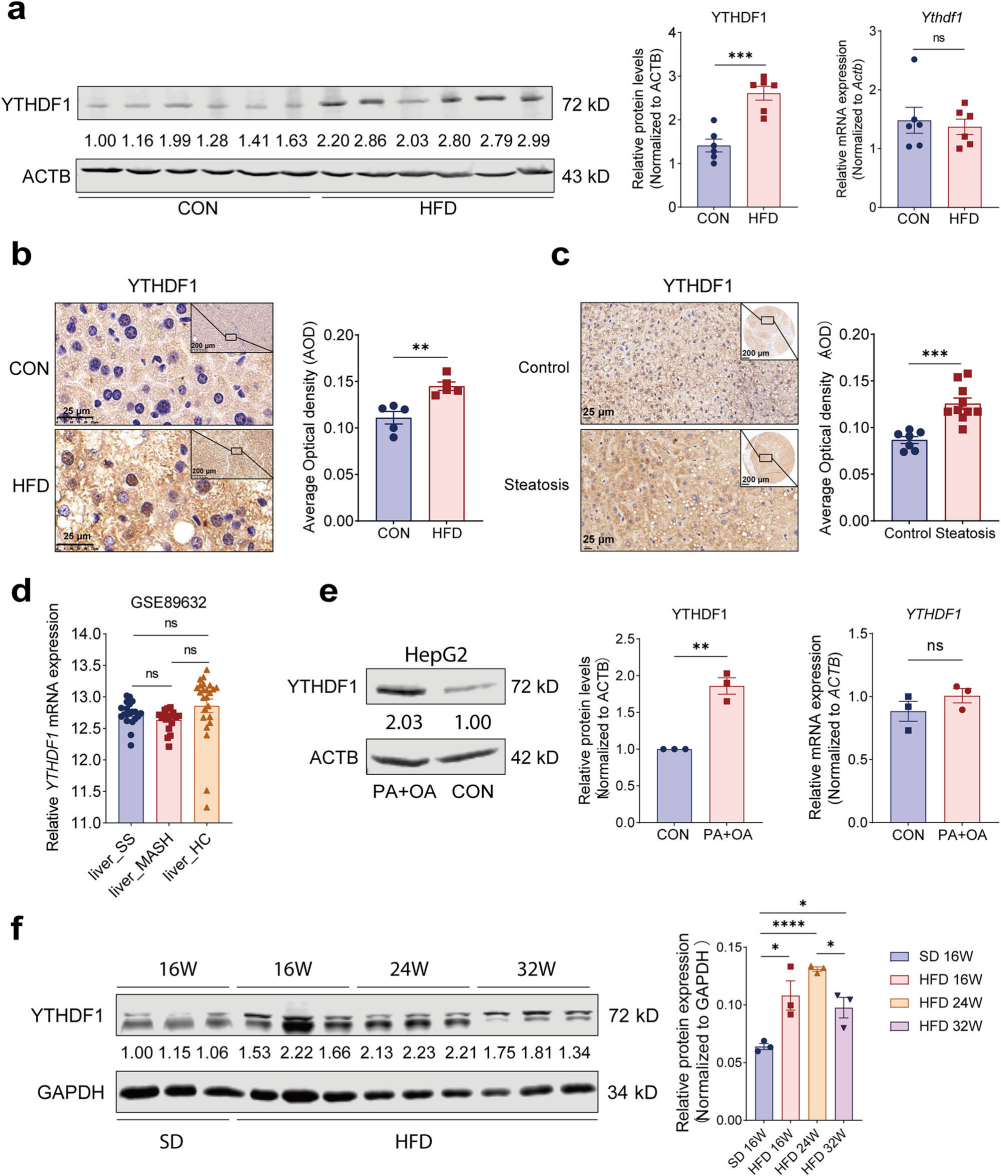
The protein level of YTHDF1 is elevated in MASLD. **a** Western blot analysis and qRT–PCR analysis of YTHDF1 in hepatic tissues from control and HFD-fed mice. Each lane contains a sample from a different mouse. *n* = 6. **b** Representative images and quantification analysis of IHC staining of YTHDF1 in liver sections from control and HFD-fed mice. *n* = 5. **c** Representative images and quantification analysis of IHC staining of YTHDF1 in human MASLD and healthy liver sections. *n* = 10 and 7, respectively. **d** Expression of YTHDF1 mRNA in human liver tissues from GSE89632. *n*_SS_ = 20, *n*_MASH_ = 19, *n*_HC_ = 24. SS, simple steatosis; HC, healthy control. **e** Western blot analysis and qRT–PCR analysis of YTHDF1 in HepG2 cells treated with PA and OA. *n* = 3, n.s. no significance. **f** Western blot and quantification analysis of YTHDF1 in hepatic tissues from HFD-fed mice for different durations. *n* = 3. The data are presented as the means ± s.e.m. n.s. no significance, ^*^P < 0.05, ^**^P < 0.01, ^***^P < 0.001 and ^****^P < 0.0001 (unpaired *t*-test).

To comprehensively understand the roles of YTHDF1 in the progression of MASLD, we detected the expression of YTHDF1 in various MASLD models. First, the protein level of hepatic YTHDF1 increased in MASLD mice fed an HFD, but the transcriptional level remained unchanged (Fig. 1a). Immunohistochemical results also revealed that YTHDF1 protein was significantly upregulated in the liver tissue of HFD-fed mice (Fig. 1b). In addition, we detected a substantial increase in the level of the YTHDF1 protein in the livers of mice with MASLD caused by the AMLND (Supplementary Fig. 1a,b) or the WD (Supplementary Fig. 1d,e), whereas the mRNA level did not change (Supplementary Fig. 1c,f).

Furthermore, IHC results revealed that YTHDF1 protein levels were elevated in human MASLD liver tissues (Fig. 1c). According to published sequencing results^26^, there was no difference in hepatic *YTHDF1* mRNA expression between healthy controls and patients with MASLD (Fig. 1d). Notably, HepG2 cells treated with palmitic acid (PA) and oleic acid (OA) to imitate MASLD in vitro^27–30^ showed elevated levels of YTHDF1 protein, although the mRNA levels remained unaltered (Fig. 1e). Intriguingly, the protein level of hepatic YTHDF1 was decreased in HFD-fed mice with advanced MASLD (Fig. 1f and Supplementary Fig. 1g,h).

Taken together, these findings indicate that YTHDF1 may be involved in the progression of MASLD and that its expression level is precisely regulated during various stages of MASLD progression.

### Hepatic loss of YTHDF1 promotes the progression of MASLD

**Fig. 2:**
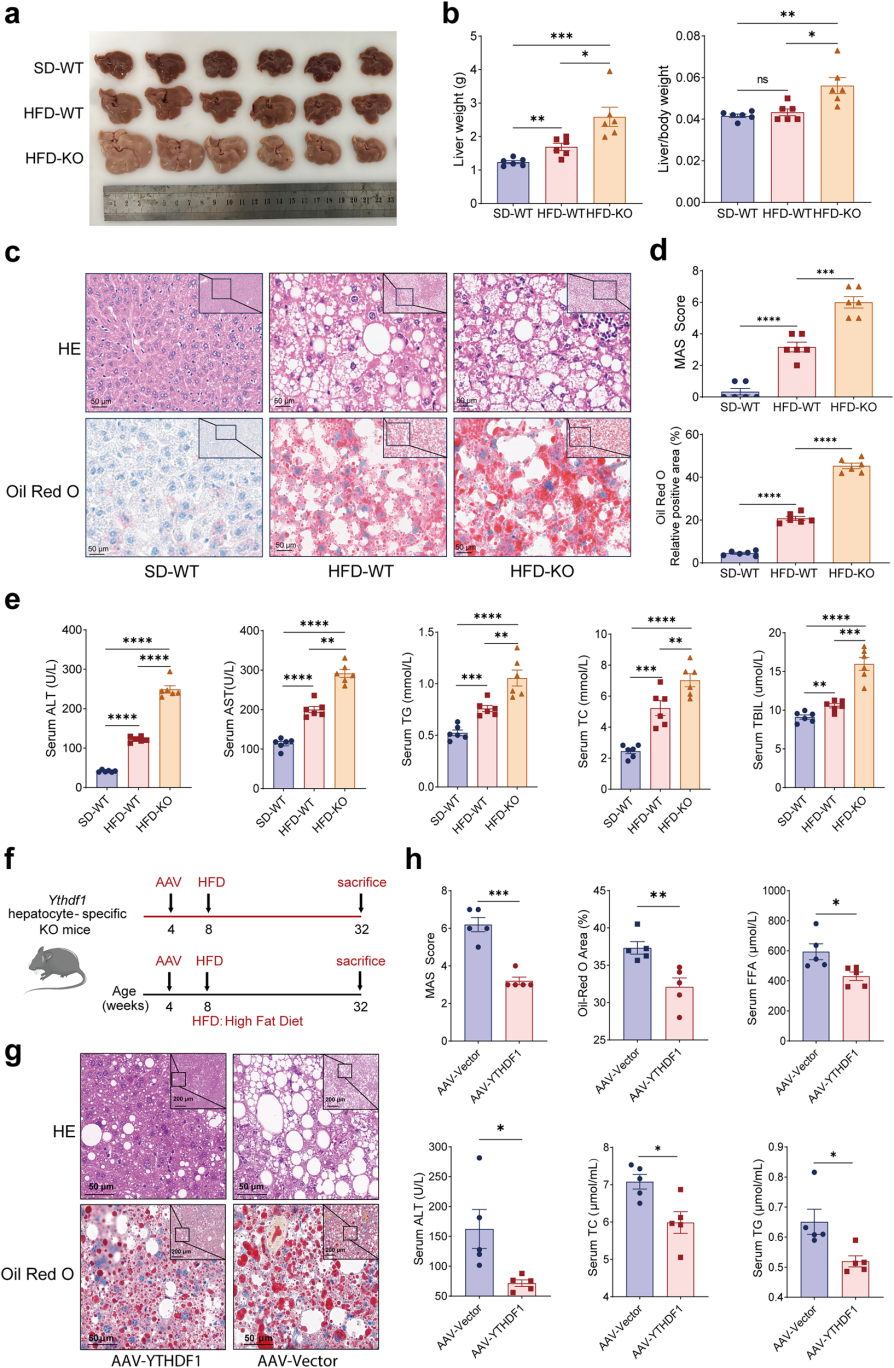
Liver-specific KO of *Ythdf1* predisposes mice to MASLD. **a** Representative images of liver from HFD-fed WT and *Ythdf1*-KO mice. *n* = 6, respectively. **b** Quantitative analysis of liver weight and liver-to-body weight ratio in HFD-fed WT and *Ythdf1*-KO mice. *n* = 6. **c** Representative images of H&E staining and representative images of Oil Red O staining. *n* = 6. **d** Quantification of the MASH scores of hepatic tissues from HFD-fed WT and *Ythdf1*-KO mice. *n* = 6. Quantification of the Oil Red O area of hepatic tissues from HFD-fed WT and *Ythdf1*-KO mice. *n* = 6. **e** The level of ALT, AST, hepatic TG, TC and TBIL of HFD-fed WT and *Ythdf1*-KO mice was measured for 16 weeks. *n* = 6, respectively. **f** Schematic diagram of the dietary feeding scheme. We randomly injected 4-week-old *Ythdf1*-KO mice with AAV-8 overexpressing YTHDF1 (AAV-YTHDF1) or control vector (AAV-Vector) through the caudal vein (*n* = 5); they were then fed an HFD for 24 weeks. **g** Representative images of H&E staining and Oil Red O staining. **h** Quantification of the MASH scores of hepatic tissues from HFD-fed YTHDF1 rescue expression in *Ythdf1*-KO mice. *n* = 5. Quantification of the Oil Red O area of hepatic tissues from HFD-fed YTHDF1 rescue expression in *Ythdf1*-KO mice. *n* = 5. The level of serum FFA, ALT, TC and hepatic TG of HFD-fed YTHDF1 rescue expression *Ythdf1*-KO mice. *n* = 5. n.s. no significance, ^*^P < 0.05, ^**^P < 0.01, ^***^P < 0.001 and ^****^P < 0.0001 (unpaired *t*-test).

To further investigate the role of YTHDF1 in MASLD, we created hepatocyte-specific *Ythdf1*-KO mice by crossing *Ythdf1*^flox/flox^ mice with albumin-Cre transgenic mice (Supplementary Fig. 2a,b). The deletion of *Ythdf1* was confirmed by western blotting and RT–PCR (Supplementary Fig. 2c). Under standard chow diet conditions, no significant differences were observed between wild-type (WT) and *Ythdf1*-KO mice in terms of liver size, liver-to-body weight ratio, hematoxylin and eosin (H&E) staining results or serum biochemical parameters, including hepatic TG content, serum TC, ALT, aspartate aminotransferase (AST) and total bilirubin (TBIL) levels, suggesting that YTHDF1 deficiency itself does not affect liver structure or basal function (Supplementary Fig. 2d–f). However, upon HFD feeding, marked differences became evident. Compared with HFD-fed WT mice, HFD-fed *Ythdf1*-KO mice exhibited a significantly enlarged liver volume, with both liver weight and liver-to-body weight ratio markedly higher than those of the control group (Fig. 2a,b). H&E staining revealed that the livers of HFD-fed *Ythdf1*-KO mice exhibited more lipid droplets and inflammatory cell infiltration compared with HFD-fed WT mice (Fig. 2c). Oil Red O staining indicated a significant increase in lipid accumulation in the livers of HFD-fed *Ythdf1*-KO mice (Fig. 2c). In addition, in the HFD group, the MASLD Activity Score (MAS) score of H&E-stained liver sections was significantly higher in KO mice compared with WT mice; quantitative analysis of Oil Red O staining also showed that the lipid deposition in the liver of KO mice was markedly higher than in their WT counterparts (Fig. 2d). Consistent with the increased lipid deposition observed in the KO mice, the levels of TG and TC were significantly elevated (Fig. 2e). In addition, the serum levels of ALT, AST and TBIL were also markedly increased in the HFD-fed *Ythdf1*-KO mice, indicating more severe liver damage (Fig. 2e). Consistently, we used short hairpin RNA (shRNA) to knock down YTHDF1 in HepG2 cells (Supplementary Fig. 3a,b) and observed increased ROS and TG levels after treatment with PA and OA (Supplementary Fig. 3c–e). Correspondingly, restoring gene expression of YTHDF1 protein in *Ythdf1*-KO mice liver cells under HFD conditions (Fig. 2f) could significantly reduce inflammatory cell infiltration and lipid accumulation (Fig. 2g,h) in mouse liver tissue. Lipid metabolism markers (free fatty acids (FFA), TC, TG and hepatic TG) and liver injury indicator (ALT) have also been partially restored (Fig. 2h). These results suggest that the hepatic loss of YTHDF1 might promote the progression of MASLD.

### Hepatic YTHDF1 deficiency promotes MASLD through peroxisome activation

**Fig. 3:**
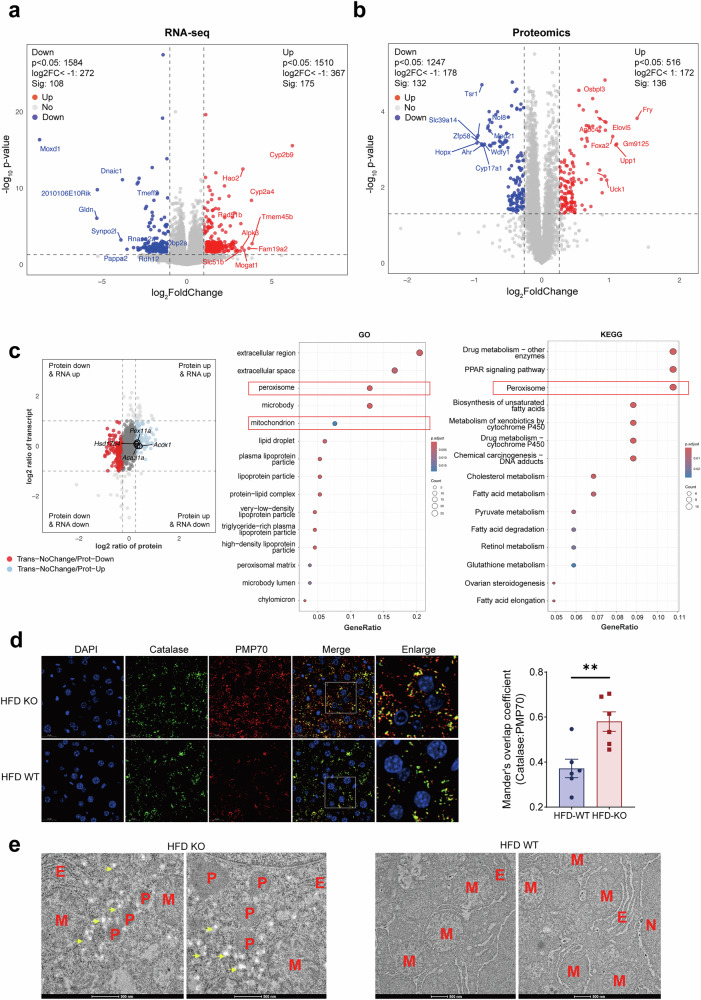
YTHDF1 loss promotes MASLD through excessive peroxisome activation. **a** Volcano plot of gene expression in the livers of HFD-fed WT and *Ythdf1*-KO mice. **b** Volcano plot of protein expression in the livers of HFD-fed WT and *Ythdf1*-KO mice. **c** Nine-quadrant graph showing the correlation between the transcriptome and proteome analyses, as well as GO and KEGG analysis revealing that the protein levels of the 137 genes increased without alterations in the mRNA levels. **d** IF staining and quantification of peroxisome marker molecules (catalase and PMP70) in the livers of HFD-fed WT and *Ythdf1*-KO mice. *n* = 6 and 7, respectively. **e** Representative TEM images of livers from HFD-fed *Ythdf1*-KO mice. P peroxisome, M mitochondrion, E endoplasmic reticulum. The data are presented as the means ± s.e.m. n.s. no significance and ^**^P < 0.01 (unpaired *t*-test). FC fold change.

To further investigate the mechanisms by which hepatic *Ythdf1* KO promotes MASLD, we performed RNA-seq and proteomic analysis of the livers of HFD-fed *Ythdf1*-KO mice. According to RNA-seq, a total of 178 and 108 genes were upregulated and downregulated, respectively (|fold change| ≥2.0, *P* < 0.05). In addition, proteomics analysis identified 136 upregulated and 132 downregulated genes (|fold change| ≥1.2, *P* < 0.05; Fig. 3a,b). Intriguingly, integrated analysis of RNA-seq and proteomics data revealed that the protein levels of most genes changed significantly (|fold change| ≥1.2, *P* < 0.05), whereas the mRNA levels remained unchanged (Fig. 3c). Specifically, the protein levels of 151 genes decreased and those of 137 genes increased without altering the mRNA levels. In the quadrant with decreased protein levels, no pathways or processes deserving special attention were enriched. Considering that YTHDF1 has traditionally been considered an important RNA m^6^A reader that promotes translation or enhances mRNA degradation^22,23^, it is difficult to explain that protein expression of 137 genes was upregulated in the liver tissues of *Ythdf1*-KO mice, while mRNA expression remained unchanged (Fig. 3c). Therefore, we further focused on these 137 genes.

**Fig. 4:**
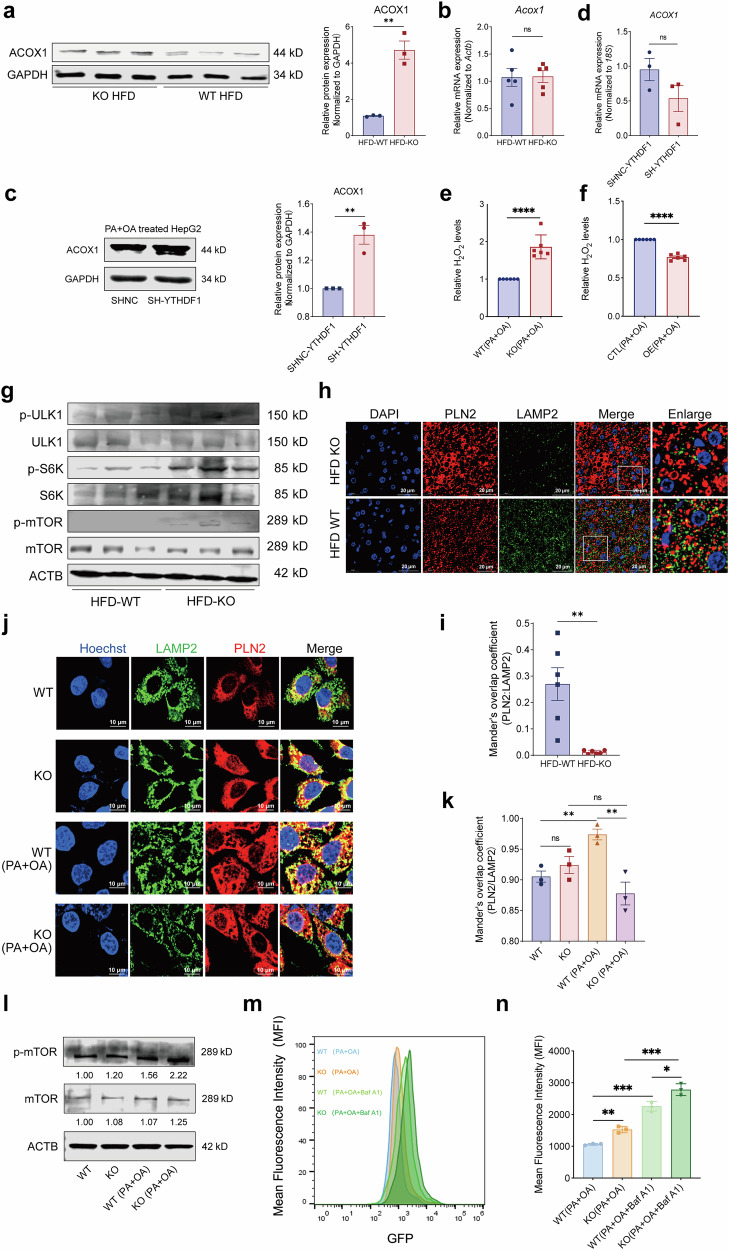
Hepatic loss of YTHDF1 increases excessive peroxisome activation by promoting ACOX1 translation under high lipid stress. **a**,**b** Western blot analysis (*n* = 3) and qRT–PCR analysis (*n* = 6 and 5, respectively) of ACOX1 in hepatic tissues from HFD-fed WT and *Ythdf1*-KO mice (HFD for 16 weeks). **c**,**d** Western blot analysis (*n* = 3) and qRT–PCR analysis (*n* = 3) of ACOX1 in YTHDF1-knockdown HepG2 cells treated with PA and OA. **e**,**f** Under PA and OA stimulation, intracellular H_2_O_2_ levels were measured in cells with YTHDF1 deficiency or overexpression as a functional readout of peroxisomal β-oxidation activity. **g** Western blot analysis of mTORC1 activation in HFD-fed WT and *Ythdf1*-KO mice (HFD for 16 weeks). *n* = 3. **h**,**i** Colocalization between lipid droplets (PLN2) and lysosomes (LAMP2) was decreased in *Ythdf1*-KO livers compared with control livers from mice fed an HFD for 16 weeks. *n* = 7 and 6, respectively. **j**,**k** Colocalization between lipid droplets (PLN2) and lysosomes (LAMP2) was decreased in *Ythdf1*-KO Hep3B cells compared with control cells treated with PA and OA. **l** Western blot analysis of mTORC1 activation in *Ythdf1*-KO Hep3B and control cells treated with PA and OA. The data are presented as the means ± s.e.m. n.s., no significance, ^**^P < 0.01 (unpaired *t*-test) and ^***^P < 0.001 (unpaired *t*-test). **m**,**n** Autophagic flux in WT and KO cells under PA- and OA-induced lipid overload was assessed using the mCherry-GFP-LC3 tandem fluorescent reporter system in combination with the lysosomal inhibitor bafilomycin A1 (Baf A1), with GFP fluorescence intensity analyzed specifically within the mCherry-positive cell population.

In the quadrant with these 137 genes, peroxisomes were enriched in both the Kyoto Encyclopedia of Genes and Genomes (KEGG) pathway and Gene Ontology (GO) analyses (Fig. 3c). Interestingly, 11 genes associated with peroxisomes were enriched in both the KEGG pathway and GO analysis (Supplementary Fig. 4a). *Pex11a*, one of the 11 genes, is associated with peroxisome proliferation^31^. The protein level of PEX11A was increased in the livers of HFD-fed *Ythdf1*-KO mice, indicating an increase in peroxisome biogenesis (Supplementary Fig. 4b). Consistent with these findings, the immunofluorescence (IF) staining and transmission electron microscopy (TEM) results revealed more peroxisomes in the livers of HFD-fed *Ythdf1*-KO mice (Fig. 3d,e). Among these 11 genes, 3 are essential for fatty acid β-oxidation in peroxisomes: ACOX1, ACCA1 and HSD17B4^13^ (Supplementary Fig. 5a). Western blot analysis revealed that HFD-fed *Ythdf1*-KO mice had elevated levels of these three proteins in their livers (Fig. 4a and Supplementary Fig. 4b). Collectively, these findings indicate that deletion of hepatic YTHDF1 enhances the development of MASLD by inducing excessive activation of peroxisomes, which may be unconventional.

### Hepatic YTHDF1 loss increases peroxisome activation via upregulation of ACOX1 protein expression under high lipid stress

ACOX1, which catalyzes the rate-limiting phase of peroxisomal β-oxidation^32^ (Supplementary Fig. 5a), was identified for further investigation. First, this study revealed that ACOX1 protein levels increased, while *ACOX1* mRNA levels remained unchanged in the livers of HFD-fed *Ythdf1*-KO mice (Fig. 4a,b) and in the livers of *YTHDF1*-knockdown HepG2 cells treated with PA and OA (Fig. 4c,d). However, elevated ACOX1 expression alone does not directly reflect changes in peroxisomal β-oxidation flux; therefore, functional validation was performed. Because ACOX1 catalysis is accompanied by the generation of H_2_O_2_, intracellular H_2_O_2_ levels were used as a functional readout of peroxisomal oxidative output. Under lipid-stimulated conditions, H_2_O_2_ accumulation was markedly increased in YTHDF1-deficient cells, whereas YTHDF1 overexpression significantly attenuated this effect (Fig. 4e,f). These results indicate that YTHDF1 not only regulates ACOX1 protein abundance but also functionally modulates peroxisome-dependent fatty acid oxidation, supporting a bona fide increase in peroxisomal β-oxidation flux under lipid stress.

In subsequent studies, the mTOR signaling pathway was overactivated in HFD-fed KO mice (Fig. 4g), and lipophagy was significantly decreased in the liver tissue of HFD-fed KO mice (Fig. 4h,i). Furthermore, we found that Hep3B cells with YTHDF1 KO stimulated by PA and OA also showed a decrease in lipophagy, while simply knocking out YTHDF1 did not alter lipophagy levels (Figs. 4j,k). Correspondingly, the mTOR signaling pathway was overactivated in PA- and OA-stimulated YTHDF1-KO Hep3B cells (Fig. 4l). To evaluate the effect of YTHDF1 deficiency on lipid autophagic flux, we used the mCherry-GFP-LC3 tandem fluorescence reporter system combined with Baf A1 treatment. Under lipid overload conditions induced by PA and OA, YTHDF1-KO cells exhibited significantly higher green fluorescent protein (GFP) fluorescence signals compared with WT cells. Following Baf A1 treatment, both WT and KO cells showed a marked shift in GFP fluorescence, suggesting persistent autophagic flux in both groups. Notably, in PA and OA combined with Baf A1-treated KO cells, the highest GFP fluorescence levels were observed, and the increase in fluorescence induced by Baf A1 was significantly greater in KO cells than in WT cells, indicating that YTHDF1 deficiency results in dysregulated autophagic flux rather than complete inhibition of autophagy (Fig. 4m,n). In summary, YTHDF1 deficiency under lipid overload conditions impairs the maturation and execution efficiency of lipid autophagy. This phenotype is strongly supported by the accumulation of PLN2-positive lipid droplets, as well as increased ACOX1 expression, mTORC activation and enhanced peroxisomal activity, suggesting that YTHDF1 modulates autophagic flux to exacerbate lipid metabolism dysregulation.

To further delineate the causal role of ACOX1 in the metabolic abnormalities associated with YTHDF1 deficiency, we performed siRNA-mediated knockdown of ACOX1 in KO cells under PA and OA stimulation. Suppression of ACOX1 expression markedly alleviated multiple pathological phenotypes induced by YTHDF1 loss. Specifically, ACOX1 knockdown significantly attenuated the hyperactivation of the mTOR signaling pathway, as indicated by reduced phosphorylation of mTOR and its downstream effector S6K. Concomitantly, the expression of the lipid droplet-associated protein PLN2 was substantially decreased, accompanied by a reduction in lipid droplet burden and intracellular lipid accumulation. In addition, ACOX1 knockdown led to a marked decrease in p62 and LC3B levels, indicating restoration of the impaired lipophagic flux in the context of YTHDF1 deficiency (Supplementary Fig. 5b,c). Collectively, these findings demonstrate that ACOX1 activity plays a pivotal role in mediating mTOR overactivation, lipophagy suppression and lipid accumulation resulting from YTHDF1 loss, thereby functionally establishing ACOX1 as a key causal driver of the associated metabolic abnormalities.

However, hepatocyte-specific YTHDF1 deletion had no effect on ACOX1 protein or mRNA levels in the mouse liver tissue under normal dietary conditions (Supplementary Fig. 5d). Similarly, in the absence of PA and OA stimulation, YTHDF1 knockdown had no effect on the protein or mRNA levels of ACOX1 in the cell line (Supplementary Fig. 5e). Under PA and OA stimulation, YTHDF1 overexpression reduced ACOX1 protein expression in cells (Supplementary Fig. 5f). Therefore, we hypothesized that high lipid stress could be a contributing factor to the increase in ACOX1 protein levels owing to decreased YTHDF1 expression.

Considering that YTHDF1, an m^6^A reader protein, promotes either mRNA degradation or protein translation^22,23^, the currently understood functions of YTHDF1 are insufficient to account for the significant increase in protein levels observed in the liver tissues of *Ythdf1*-KO mice and no changes in their mRNA levels (especially ACOX1). This means that YTHDF1 may exert regulatory effects in a new way. We further used SILAC quantitative mass spectrometry (SILAC–MS/MS) technology to identify potential interacting proteins of YTHDF1. It is interesting that those experiments demonstrated that YTHDF1 interacts with proteins that are part of the SG complex or mitochondrial components (Supplementary Fig. 6a). In addition, IF analysis using antibodies against YTHDF1 and G3BP1, which is a core protein necessary for SG formation and can serve as a SG marker, revealed their spatial colocalization (Supplementary Fig. 6b). Furthermore, we observed a substantial decrease in the quantity of SGs in YTHDF1-silenced HepG2 cells under high lipid stress (Supplementary Fig. 6c). Collectively, these results suggest that YTHDF1 might influence SG formation during high lipid stress.

Next, we found that *ACOX1* mRNA could be sequestered within SGs and that reducing the expression of YTHDF1 decreased the sequestration of *ACOX1* mRNA in SGs (Supplementary Fig. 6d). Subsequent RIP–qPCR demonstrated that YTHDF1 depletion significantly decreased the sequestration of *ACOX1* mRNA in SGs (Supplementary Fig. 6e). To verify the necessity of SGs in the regulation of ACOX1 by YTHDF1, we first compared the differences in SGs formation between the si-G3BP1 treatment group and the control group using western blotting and IF assays. The results showed that knockdown of G3BP1 significantly reduced the number of SGs, and this finding was further validated by quantitative analysis of the SG marker proteins TIA-1 and G3BP1. We further knocked down G3BP1 to reduce SGs to confirm that SGs are necessary for the ability of YTHDF1 to regulate ACOX1. As expected, ACOX1 protein levels increased in response to SG reduction; this effect was particularly pronounced when SGs levels were decreased in YTHDF1-overexpressing cells during elevated lipid stress (Supplementary Fig. 6f,g). Subsequently, YTHDF1-knockdown HepG2 cells were transfected with a Flag-YTHDF1 expression plasmid (YTHDF1-WT) and a Flag-YTHDF1 mutant plasmid (YTHDF1-Mut, K395A and Y397A) that were incapable of binding to RNA^33^. We discovered that under high lipid stress, overexpression of YTHDF1-WT, but not of YTHDF1-Mut, decreased ACOX1 protein levels (Supplementary Fig. 6h). These findings collectively suggest that YTHDF1 regulates ACOX1 protein levels by promoting SG formation in MASLD, which is dependent on the RNA-binding domain of YTHDF1.

### YTHDF1 deletion promotes MASLD by disrupting mitochondrial homeostasis

**Fig. 5:**
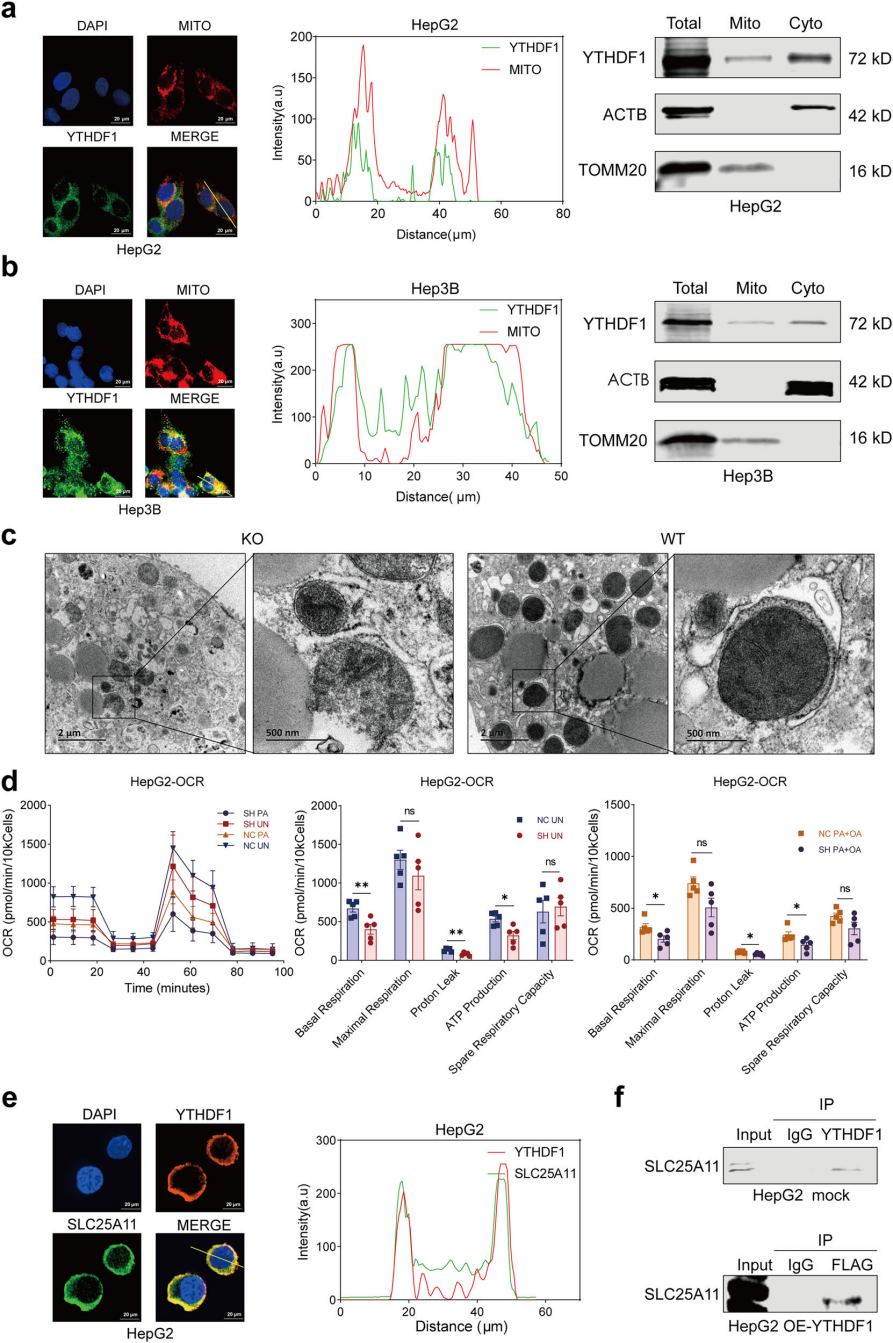
YTHDF1 can be localized in mitochondria and bind to SLC25A11. **a** IF staining and mitochondrial fractionation assays showing the localization of YTHDF1 in the mitochondria of HepG2 cells. **b** IF staining and mitochondrial fractionation assays showing the localization of YTHDF1 in the mitochondria of Hep3B cells. **c** Representative TEM images of primary hepatocytes from *Ythdf1*-KO (left) and WT mice (right) treated with PA and OA. **d** OCR in YTHDF1-silenced HepG2 cells treated with PA and OA determined by Seahorse. **e** IF staining showing the colocalization between YTHDF1 and SLC25A11 in HepG2 cells. **f** Co-IP was conducted with HepG2 whole-cell extracts to confirm that YTHDF1 interacted with SLC25A11. The data are presented as the means ± s.e.m. n.s. no significance, ^*^P < 0.05 and ^**^P < 0.01 (unpaired *t*-test).

SILAC–MS/MS data also revealed that YTHDF1 interacts with at least five mitochondrial proteins, including SLC25A11, SLC25A24, SLC25A6, TMEM70 and ALDH1B1 (Supplementary Fig. 6a). Consistent with this finding, IF and mitochondrial fractionation assays confirmed that YTHDF1 was located in the mitochondria of HepG2 (Fig. 5a), Hep3B (Fig. 5b) and THLE-2 (Supplementary Fig. 7a) cells. TEM results revealed that PA- and OA-treated primary hepatocytes derived from *Ythdf1*-KO mice had more severely damaged mitochondria, characterized by fragmentation, swelling and an absence of cristae (Fig. 5c). The Seahorse assay revealed a decrease in the OCR in YTHDF1-knockdown HepG2 cells (Fig. 5d) and immortalized hepatocyte THLE-2 cells (Supplementary Fig. 7b). Interfering with YTHDF1 resulted in lower basal respiration, proton leakage and ATP generation in HepG2 cells (Fig. 5d) and THLE-2 cells (Supplementary Fig. 7b), which were also significantly lower in *YTHDF1*-knockdown HepG2 (Fig. 5d) and THLE-2 cells (Supplementary Fig. 7b) treated with PA and OA. These results indicated that YTHDF1 could be localized in mitochondria, and its absence might lead to mitochondrial dysfunction.

SILAC–MS/MS identified SLC25A11 as the top interactor of YTHDF1 (Supplementary Fig. 6a). SLC25A11 is a recognized transporter that transfers glutathione (GSH) from the cytoplasm to the mitochondria, maintaining GSH levels and reducing ROS generation^34^. Our findings revealed that YTHDF1 interacted with SLC25A11, as demonstrated by IF experiments in HepG2 (Fig. 5e), Hep3B (Supplementary Fig. 7c) and THLE-2 cells (Supplementary Fig. 7d). Co-IP analysis also confirmed the interaction between YTHDF1 and SLC25A11 in HepG2 cells (Fig. 5f). In addition, SLC25A11 protein levels were lower in HFD-fed *Ythdf1*-KO mice than in WT mice (Supplementary Fig. 7e). In addition, KEGG pathway analysis of the metabolites enriched in the metabolomic data revealed an enrichment in GSH metabolism (Supplementary Fig. 7f), and the GSH levels were elevated in HFD-fed *Ythdf1*-KO mice, which is consistent with the metabolomic results (Supplementary Fig. 7g,h).

**Fig. 6:**
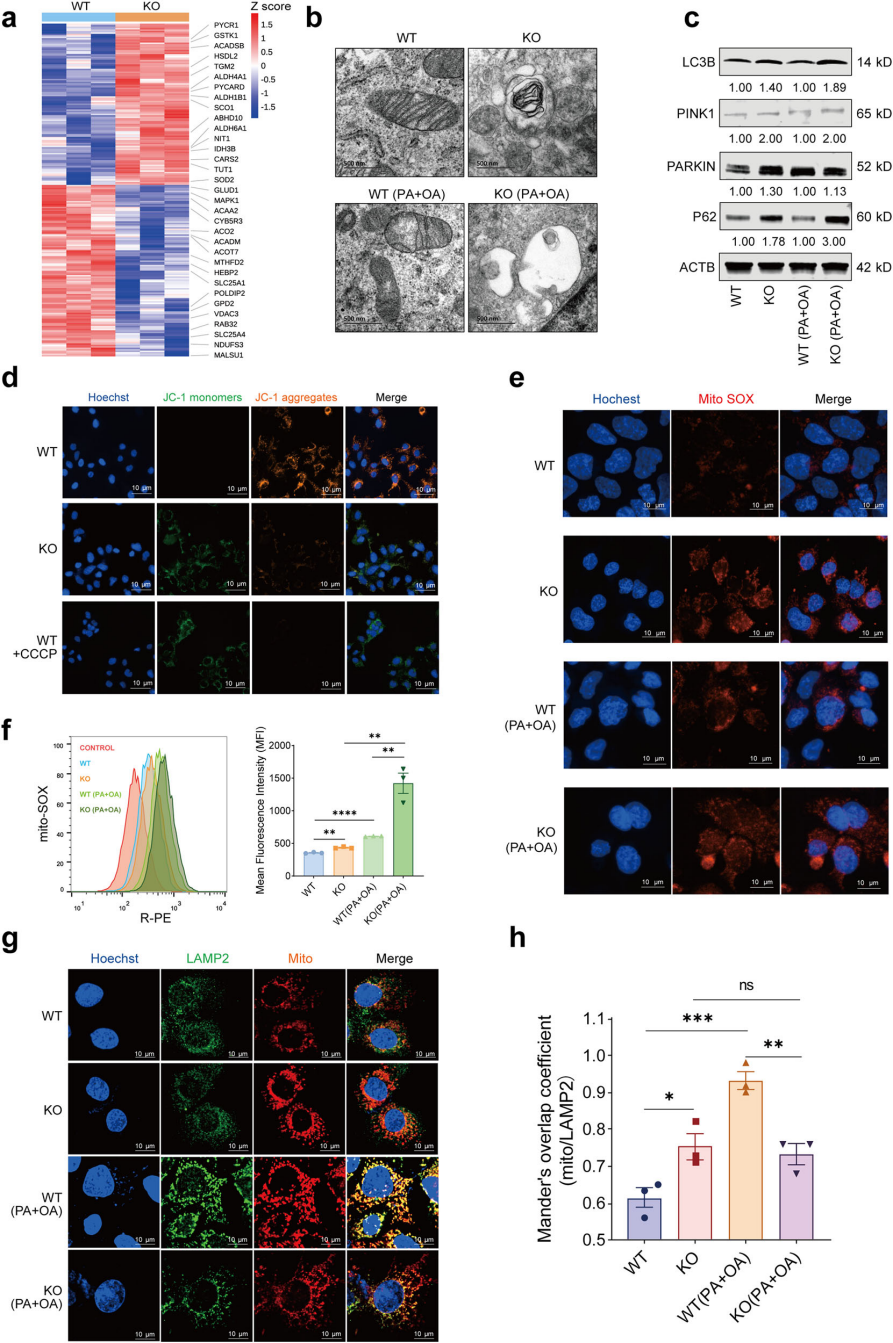
YTHDF1 deficiency disrupts mitochondrial homeostasis. **a** Heat map presenting differentially expressed proteins in YTHDF1-KO Hep3B (KO) and control (WT) cells (*n* = 4). Mitochondria-related proteins were labeled. **b** Representative TEM images of YTHDF1-KO Hep3B (KO) and control (WT) cells treated with PA and OA. **c** Western blot analysis of autophagy markers in YTHDF1-KO Hep3B (KO) and control (WT) cells treated with PA and OA. **d** Images of YTHDF1-KO Hep3B (KO) and control (WT) cells stained with JC-1 probe for 30 min. Scale bar, 10 μm. CCCP is a positive control for inducing a decrease in mitochondrial membrane potential. **e**,**f** Confocal fluorescence images (**e**) and flow cytometry quantitative analysis (**f**) of Mito-ROS production in YTHDF1-KO Hep3B (KO) and control (WT) cells using mitochondrial superoxide indicator of MitoSOX Red probe. Data are shown as the mean ± s.d. (*n* = 3). **g**,**h** Colocalization between mitochondrion (MitoTracker Deep Red FM) and lysosomes (LAMP2) was decreased in YTHDF1-KO Hep3B cells compared with control cells treated with PA and OA. The data are presented as the means ± s.e.m. n.s., no significance, ^*^P < 0.05, ^**^P < 0.01, ^***^P < 0.001 (unpaired *t*-test).

To further clarify the role of YTHDF1 in mitochondrial metabolism, we constructed Hep3B cells with YTHDF1 gene KO. Compared with the control Hep3B cells (WT), the proteomic results showed that 33 mitochondrial-related proteins were differentially expressed in KO cells (Fig. 6a and Supplementary Tables 1 and 2). The observation results of TEM showed that the number of autophagosomes in the KO cells increased regardless of whether there was PA and OA stimulation (Fig. 6b). The results of western blot showed that autophagy markers were significantly upregulated in KO cells, which also confirmed this result (Fig. 6c). Next, we conducted JC-1 staining to measure mitochondrial membrane potential (Δ*ψ*_m_) and MitoSox Red staining to assess mitochondrial ROS levels. It has been known that in normal mitochondrion, JC-1 aggregates in the mitochondrial matrix to form polymers emitting a strong red fluorescence, whereas in an unhealthy mitochondria, it can only exist in the cytoplasm in the form of monomers, emitting a green fluorescence. As shown in Fig. 6d, the red fluorescence basically disappeared and was replaced by a strong green fluorescence in KO cells, indicating that the mitochondrial membrane potential is almost completely dissipated. In addition, a substantial increase in mitochondrial ROS content in KO cells was observed (Figs. 6e,f). Collectively, these results indicate that KO of YTHDF1 in Hep3B cells results in mitochondrial dysfunction. We further examined the colocalization of mitochondrial (MitoTracker Deep Red FM) and lysosomal (LAMP2) markers. It was found that in Hep3B cells treated with PA and OA, the colocalization signals of mitochondrial and lysosomal markers were significantly reduced when YTHDF1 was knocked out (Fig. 6g,h). These results indicated that the maturation stage of mitochondrial autophagosomes was hindered in YTHDF1-KO Hep3B cells.

Taken together, these findings suggest that YTHDF1 deletion may reduce the protein expression of SLC25A11, which in turn may restrict the supply of GSH to the mitochondria, thereby promoting the progression of accumulation of mitochondrial ROS, which may initiate the process of mitochondrial autophagy. However, the absence of YTHDF1 reduces the fusion of mitochondrial autophagosomes and lysosomes, thereby disrupting autophagic flux.

### Methylation of the YTHDF1 protein decreases its stability

**Fig. 7:**
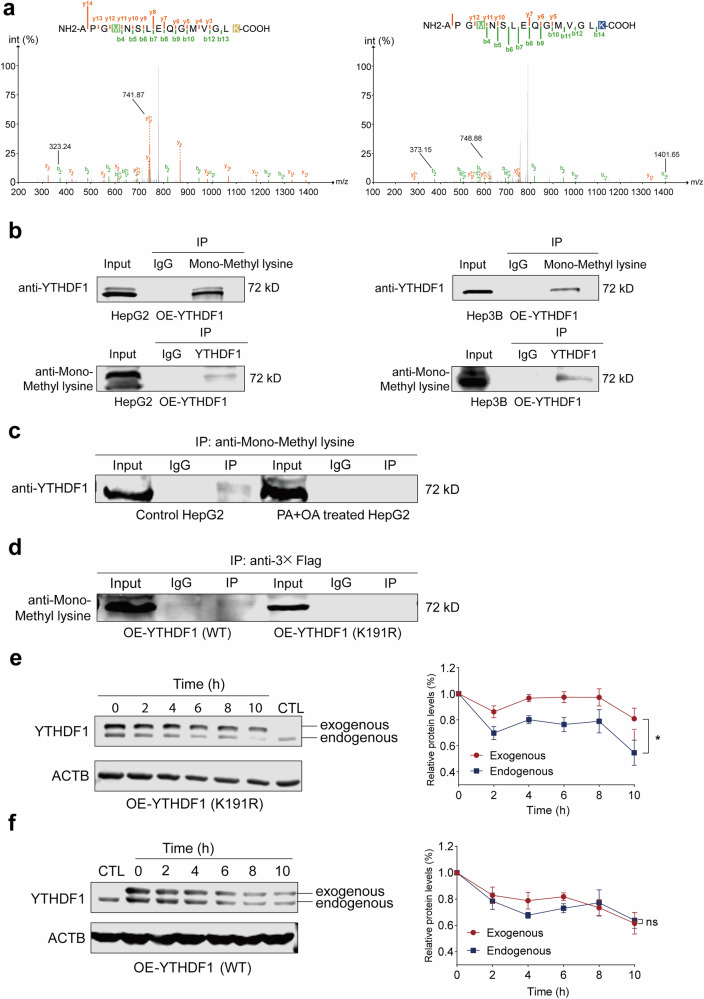
Methylation of the YTHDF1 protein decreases its stability. **a** SILAC–MS/MS results showing that the K191 site was monomethylated in YTHDF1. **b** Co-IP was conducted with HepG2 and Hep3B whole-cell extracts to confirm the methylation of YTHDF1. **c** Co-IP results showing the methylation level of YTHDF1 under high lipid stress conditions. **d** Co-IP results showing that YTHDF1 K191R prevented methylation. **e** Western blot analysis of exogenously expressed YTHDF1-K191R mutants and endogenously expressed YTHDF1. The quantitative data of strip grayscale values are displayed on the right. **f** Western blot analysis of exogenously and endogenously expressed YTHDF1. The quantitative data of strip grayscale values are displayed on the right. The data are presented as the means ± s.e.m. n.s. no significance and ^*^P < 0.05 (unpaired *t*-test).

Previous studies have shown that a lack of *S*-adenosylmethionine (SAM), which is an important supplier of methyl groups for the methylation of many substances, such as DNA and proteins, leads to hypomethylation of DNA and proteins during the progression of MASLD^35,36^. In this study, SILAC–MS/MS revealed that the K191 lysine site is monomethylated in YTHDF1, which has not been previously reported (Fig. 7a). Furthermore, IP analysis confirmed the existence of YTHDF1 methylation using a specific pan-monomethyl lysine antibody in HepG2 and Hep3B cell lines (Fig. 7b). Interestingly, this study revealed that the methylation level of YTHDF1 decreased under high lipid stress conditions (Fig. 7c).

To determine whether methylation of YTHDF1 affects its stability, arginine was substituted for the K191 lysine residue to generate the YTHDF1 mutant. This preserves the positive charge and prevents methylation, thereby simulating the nonmethylated version of YTHDF1 (Fig. 7d). We subsequently transfected HepG2 cells with the Flag-YTHDF1 expression plasmid (YTHDF1-WT) or the Flag-YTHDF1 mutant plasmid (YTHDF1- K191R). The exogenous YTHDF1^K191R^ mutant degraded more slowly and had stronger protein stability than the endogenous YTHDF1 (Fig. 7e), whereas the exogenous YTHDF1-WT decayed in the same manner as the endogenous YTHDF1, with no significant difference in protein stability (Fig. 7f). Taken together, these results indicate that YTHDF1 could be methylated at Lys191, and that methylation might decrease the protein stability of YTHDF1.

## Discussion

In the present study, we investigated the potential protective function of YTHDF1 during MASLD progression. The protein level of YTHDF1 increases in individuals and in various mouse models and cell models of MASLD. However, the expression levels of YTHDF1 protein might decrease in the late stage of MASLD progression (Fig. 1f and Supplementary Fig. 1g,h). Furthermore, RNA-seq and proteomic analysis of the livers of HFD-fed *Ythdf1*-KO mice revealed that YTHDF1 loss promoted MASLD through excessive peroxisome activation and impaired mitochondrial function. In addition, YTHDF1 can be methylated at Lys191, which may reduce its stability.

YTHDF1 is recognized as an important m^6^A reader that increases translation and enhances mRNA degradation^22,23,37^. Our findings indicate that the deletion of YTHDF1 resulted in an increase in the protein level, but not the mRNA level, of 137 genes under elevated lipid stress, which is inconsistent with current reports. An intriguing observation from the SILAC–MS/MS data was that YTHDF1 interacted with SG component proteins and mitochondrial proteins, suggesting that YTHDF1 is crucial for the functions of SGs and mitochondria. Furthermore, we discovered that *ACOX1* mRNA could be sequestered inside SGs and that decreased YTHDF1 expression reduced the sequestration of *ACOX1* mRNA in SGs. YTHDF1 deletion increased ACOX1 protein levels by lowering *ACOX1* mRNA sequestration in SGs, thereby increasing peroxisome activation. In addition, YTHDF1 is localized in the mitochondria and interacts with SLC25A11, affecting ROS production and mitochondrial GSH transport. These findings suggest that YTHDF1 may play a role in the evolution of MASLD by modulating peroxisome and mitochondrial functions in an unconventional manner.

Peroxisomes are critical for lipid metabolism, including ether lipid production, bile acid synthesis, α-oxidation of branched-chain fatty acids and β-oxidation of very-long-chain fatty acids^38^. It has been suggested that animals resistant to diet-induced steatosis and obesity have higher peroxisomal β-oxidation rates and that reductions in hepatic β-oxidation cause hepatic steatosis^39,40^. ACOX1, one of the 11 candidate peroxisome-related genes, was selected for further study because it catalyzes the peroxisomal β-oxidation step, which is rate limiting^41^. In addition, acetyl-CoA is produced by ACOX1-mediated peroxisomal β-oxidation, which inhibits lipophagy and increases mTORC1 levels, ultimately resulting in hepatic steatosis^13^. Furthermore, the ROS generated by ACOX1-mediated peroxisomal β-oxidation stabilizes PEX2, which in turn reduces lipolysis and increases adipose triglyceride lipase breakdown, thereby increasing lipid accumulation in MASLD^14^. Consistent with these findings, our results revealed that the mTOR signaling pathway was overactivated and that lipophagy was significantly weaker in HFD-fed *Ythdf1*-KO mice. Importantly, we observed that *ACOX1* mRNA could be sequestered in SGs and that *ACOX1* mRNA sequestration in SGs was diminished by reducing YTHDF1 expression. ACOX1 was identified as a downstream regulator of YTHDF1, which influences peroxisome function through SGs during high lipid stress.

Mitochondrial dysfunction is closely associated with the development and progression of hepatic steatosis and MASLD^11^. Previous research has demonstrated that deletion of YTHDF1 in hepatocytes hampers mitochondrial function and elevates oxidative stress^42^. Furthermore, we discovered that YTHDF1 could be localized in mitochondria and that YTHDF1 depletion impairs mitochondrial OXPHOS and restricts the delivery of GSH to mitochondria by reducing the expression of SLC25A11. During the progression of MASLD, the expression level of YTHDF1 gradually decreases, which probably contributes to mitochondrial dysfunction and the gradual accumulation of ROS. Further investigation is needed to better understand the mechanisms by which YTHDF1 affects mitochondrial OXPHOS and SLC25A11 expression.

Protein methylation is a type of post-translational modification (PTM) that regulates the process of protein subunit or whole protein degradation through the covalent addition of functional groups or proteins, including phosphorylation, glycosylation, ubiquitination, nitrosylation, methylation, acetylation and lipidation, thereby affecting almost all aspects of normal cell biology and pathogenesis^43,44^. O-GlcNAcylation of YTHDF1 modifies its cytosolic position and induces crosslinking with other PTMs of RNA and proteins^45,46^. Notably, we discovered that YTHDF1 can be methylated at Lys191 and that methylation may decrease YTHDF1 protein stability, which has not been previously reported. According to an earlier study, the methylation levels of DNA and protein in the liver tissues of patients with MASLD are often lower than those in normal liver tissues because of the decrease in the level of the methyl donor SAM^35^. This investigation consistently revealed that the methylation level of YTHDF1 decreased under high lipid stress conditions, whereas its protein level increased. The downregulation of YTHDF1 protein levels in the late stages of MASLD may be linked to an increase in methylation levels; however, this process is still being investigated.

In conclusion, our work revealed that the expression of YTHDF1 protein was increased in MASLD, which prevented disease progression by controlling SG sequestration of *ACOX1* mRNA and maintaining mitochondrial homeostasis. This study elucidated the mechanism of YTHDF1 in the progression of MASLD and provided theoretical support for the development of therapeutic drugs targeting YTHDF1 and its related signaling pathways, as well as the optimization of clinical treatment decisions for MASLD.

## Supplementary information

Supplementary Information

Supplementary Table 1

Supplementary Table 2
