# Supplementary material for: Noncanonical function of epigenetic reader YTHDF1 inhibits MASLD progression by maintaining peroxisomes and mitochondrial homeostasis
Source: Exp Mol Med. 2026 Apr 10;58(4):1172–86. doi: 10.1038/s12276-026-01686-3 (PMC13144363; doi:10.1038/s12276-026-01686-3)
Supplement: Supplementary file 1 — Supplementary Information [file 12276_2026_1686_MOESM1_ESM.pdf]

Epigenetic reader YTHDF1 noncanonical function maintains peroxisomes and mitochondrial homeostasis: inhibiting MASLD progression

Chenyang Mu[1, 2, #], Jian Tan[1, #], Yuefan Wang[3, 6, #], Haozan Yin[1], Zhihui Dai[1], Zenghan Wang[1], Sijie Wang[1, 2], Cuitong He[1], Dongyang Ding[3], Zhichao Zhang[3], Hui Liu[3], Fu Yang[1, 4, 5]

[1]Department of Medical Genetics, Naval Medical University, Shanghai, China.

[2]School of Health Science and Engineering, University of Shanghai for Science and Technology, Shanghai, China.

[3]Third Department of Hepatic Surgery, Eastern Hepatobiliary Surgery Hospital, Naval Medical University, Shanghai, China.

[4]Shanghai Key Laboratory of Medical Bioprotection, Shanghai, China.

[5]Key Laboratory of Biological Defense, Ministry of Education, Shanghai, China.

[6]Research Institute of General Surgery, Nanjing University School of Medicine or General Hospital of Eastern Theater Command, PLA, Nanjing, Jiangsu, China.

[#]Contributed equally.

Correspondence: Fu Yang (yangfusq1997@smmu.edu.cn) and Hui Liu (liuhuigg@hotmail.com).

Short title: Non-traditional mechanism of YTHDF1 in MASLD

Supplementary figure legends

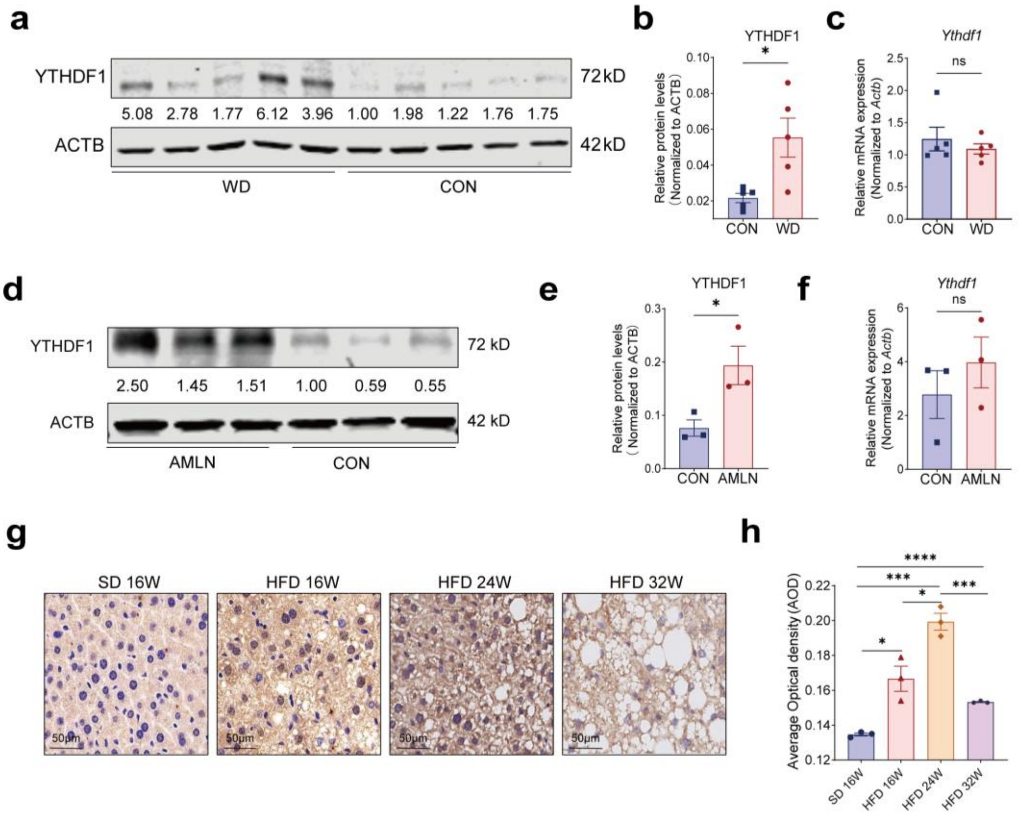

Supplementary Figure 1. YTHDF1 protein level is elevated in MASLD induced by WD and AMLND. (a) Western blot analysis of YTHDF1 in hepatic tissues from control and WD-fed mice. Each lane contains a sample from a different mouse. (b) Quantification of results in panel A. n=5. (c) Quantitative real-time PCR analysis of *Ythdf1* mRNA levels in hepatic tissues from control and WD-fed mice. n=5. (d) Western blot analysis of YTHDF1 in hepatic tissues from control and AMLN-fed mice. Each lane contains a sample from a different mouse. (e) Quantification of results in panel D. n=3. (f) Quantitative real-time PCR analysis of *Ythdf1* mRNA levels in hepatic tissues from control and AMLN-fed mice. n=3. The data are presented as the means ± SEMs. ns, no significance, and $*p < 0.05$ (unpaired t test). Immunohistochemical staining (g) and quantification analysis (h) of YTHDF1 in hepatic tissues from HFD-fed mice for different durations. n = 3. The data are presented as the means ± SEMs. $**p < 0.01$,

***p < 0.001 (unpaired t test).

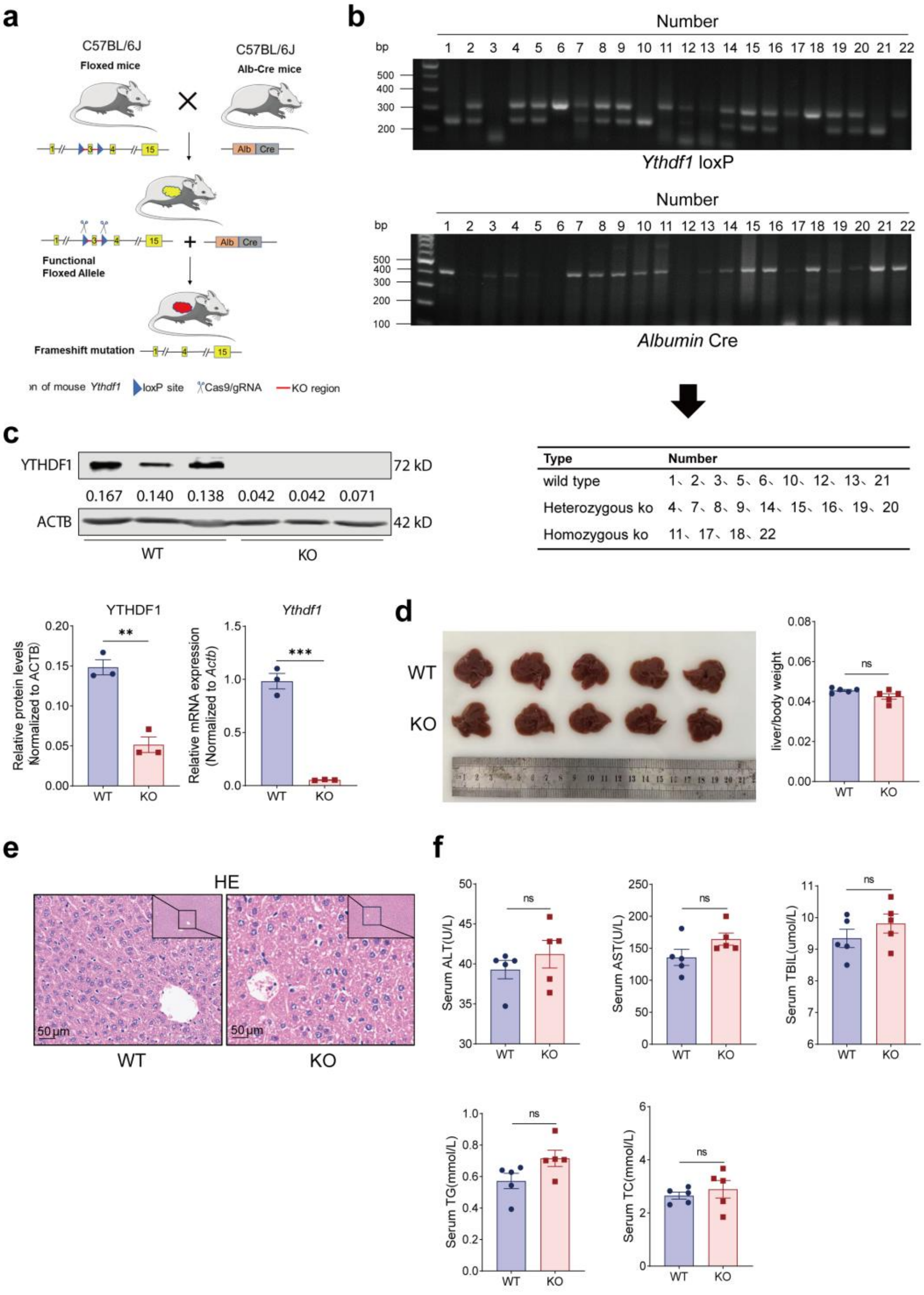

Supplementary Figure 2. **Construction and validation of liver-specific *Ythdf1*-knockout mice.** (a) Schematic diagram of the generation of hepatocyte-specific Ythdf1-knockout mice. (b) Genotype identification of transgenic mice. (c) Western blot and quantitative real-time PCR analysis of YTHDF1 in YTHDF1-knockdown cell lines.

n=3. (d) Representative images of livers from wild-type and knockout mice, along with statistical analysis of the liver-to-body weight ratio. (e) Hematoxylin and eosin (H&E) staining of liver tissues from wild-type and knockout mice. (f) The level of alanine aminotransferase (ALT), aspartate aminotransferase (AST), hepatic triglyceride (TG), total cholesterol (TC), and total Bilirubin (TBIL) of WT and *Ythdf1*-KO mice.

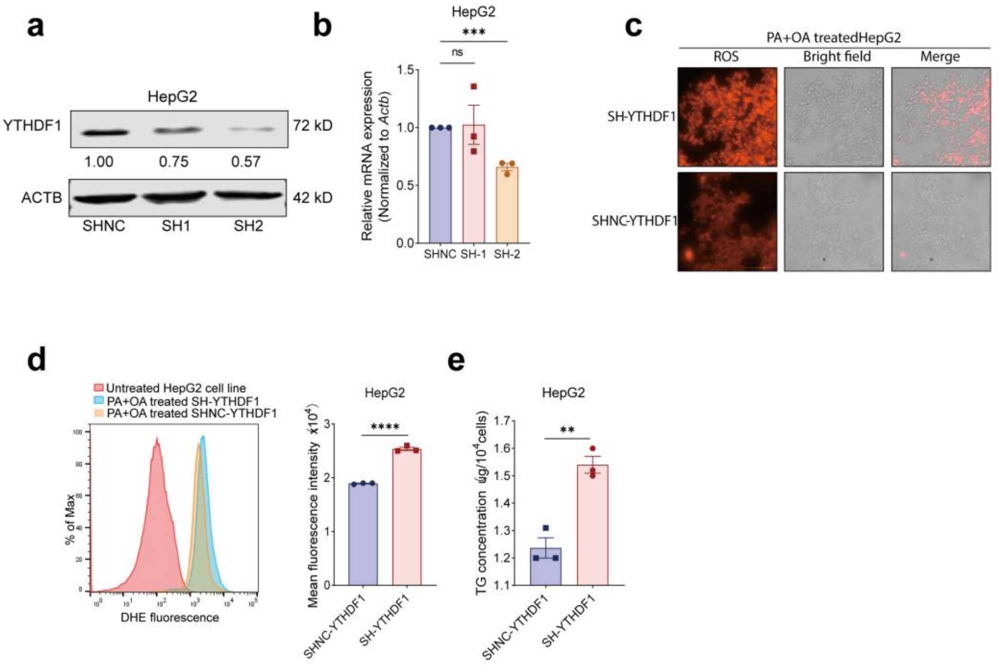

Supplementary Figure 3. **Construction and validation of YTHDF1 knockout cell lines.** (a) Western blot analysis of YTHDF1 expression in knockdown cell lines. (b) Quantification of results in panel A. n=3. (c-e) IF staining and flow cytometry analysis of ROS in YTHDF1-knockdown Hep G2 cells treated with PA and OA, as well as Triglyceride content in YTHDF1-knockdown Hep G2 cells treated with PA and OA (right). n =3. The data are presented as the means ± SEMs. ns, no significance, $**p < 0.01$, $***p < 0.001$, and $****p < 0.0001$ (unpaired t test).

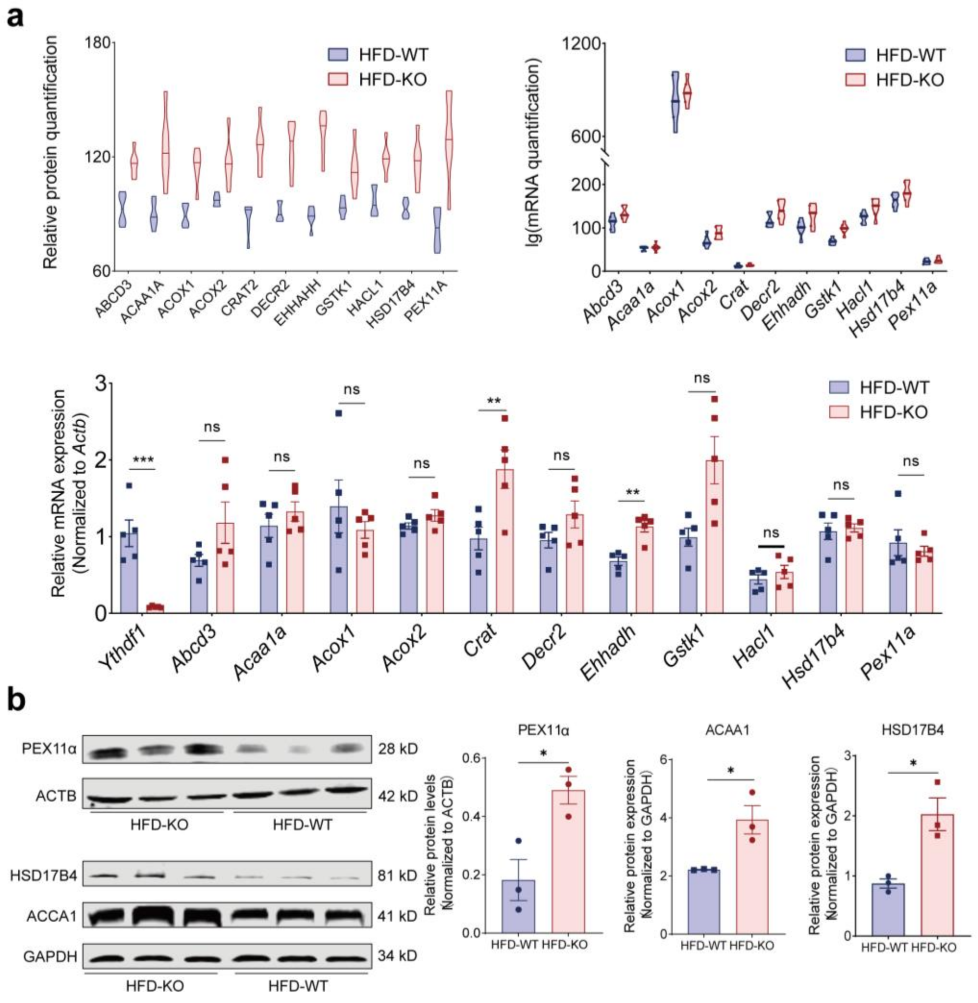

Supplementary Figure 4. **RNA-seq and proteomic data identify peroxisome as a potential target of YTHDF1.** (a) Relative protein and mRNA quantification of 11 genes linked with peroxisome in proteomic data and RNA-seq data, respectively, as well as quantitative real-time PCR analysis of 11 genes linked with peroxisome in hepatic tissues from HFD-fed WT and *Ythdf1*-KO mice (n=5 and 6, respectively). (b) Western blot analysis of PEX11α, HSD17B4, and ACCA1 in hepatic tissues from HFD-fed WT and *Ythdf1*-KO mice. n = 3. The data are presented as the means ± SEMs. ns, no significance, *p < 0.05, **p < 0.01, and ***p < 0.001 (unpaired t test).

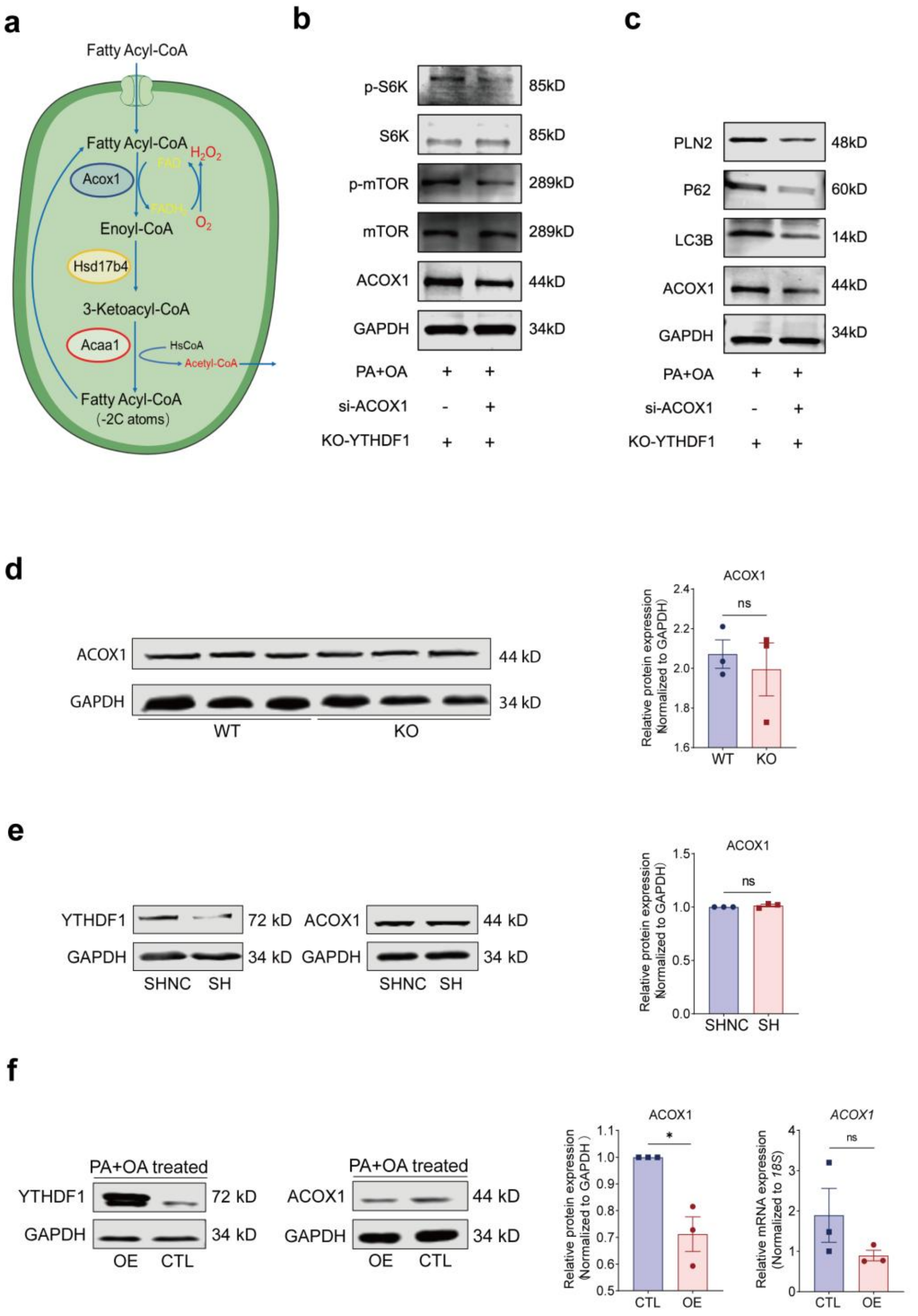

Supplementary Figure 5. **YTHDF1 knockdown had no effect on ACOX1 expression in the absence of stress.** (a) Schematic of peroxisomal β-oxidation. (b-c) Under PA and OA treated conditions, KO cells were subjected to siRNA-mediated knockdown of ACOX1. The expression levels of mTOR signaling–related proteins (mTOR and p-S6K), the lipid droplet–associated protein PLN2, and the autophagy-

related proteins p62 and LC3B were examined by immunoblotting. (d) Western blot analysis of ACOX1 in hepatic tissues from standard diet (SD)-fed WT and *Ythdf1*-KO mice. n=3. (e) Western blot analysis of YTHDF1 and ACOX1 in YTHDF1-knockdown cell lines. n=3. (f) Western blot analysis of YTHDF1 and ACOX1 in cell lines overexpressing YTHDF1 after PA and OA treatment, as well as quantitative real-time PCR analysis of ACOX1 in cell lines overexpressing YTHDF1 after PA and OA treatment. n=3. The data are presented as the means ± SEMs. ns, no significance, *$p < 0.05$, and **$p < 0.01$ (unpaired t test).

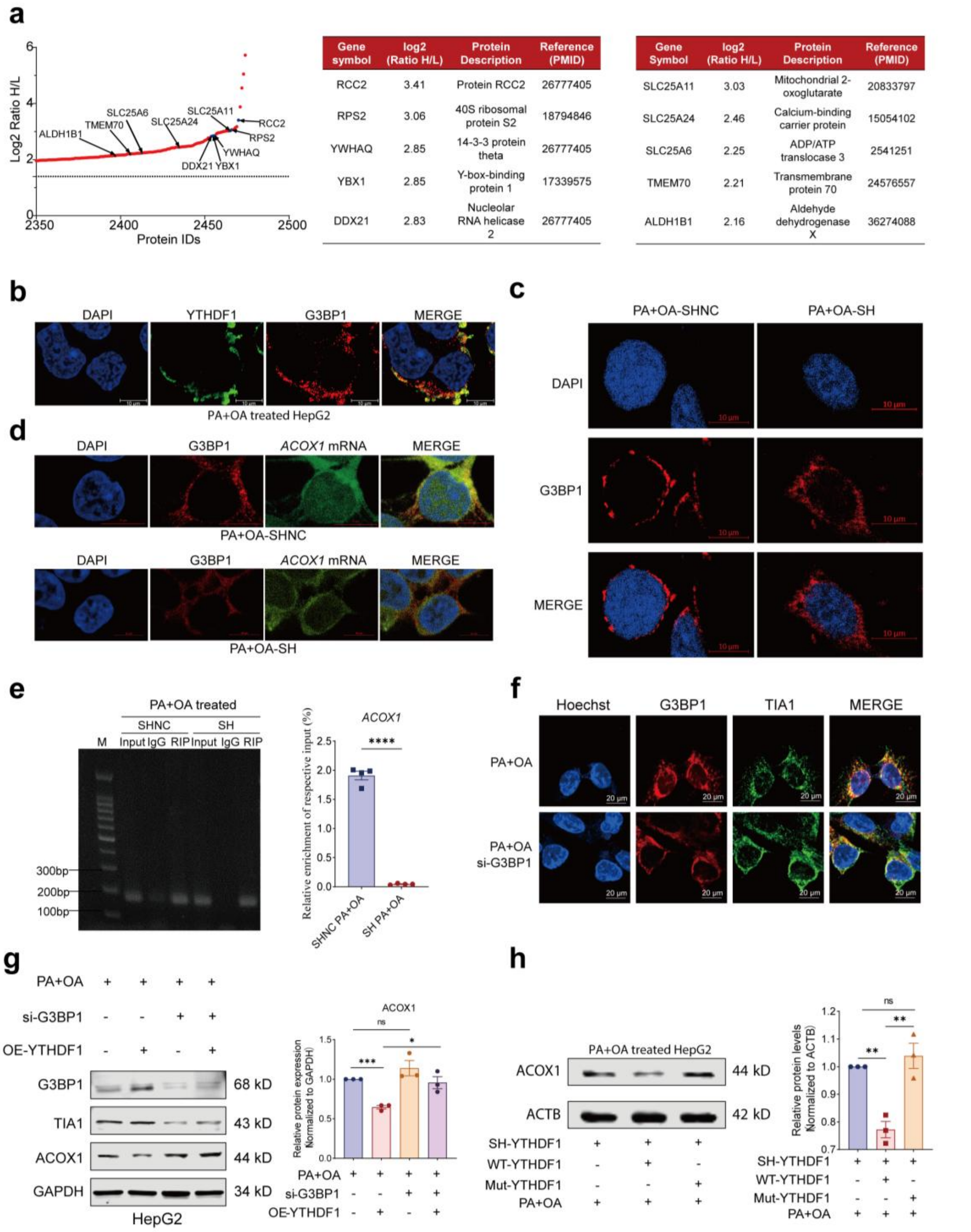

Supplementary Figure 6. YTHDF1 regulates ACOX1 expression by facilitating the formation of SGs in MASLD. (a) Potential YTHDF1-interacting proteins identified by SILAC-MS and representative SG component proteins or mitochondria-associated proteins that interact with YTHDF1. (b) IF staining of SG (G3BP1) and YTHDF1 in HepG2 cells under MASLD stress. (c) IF image showing the number of SGs in HepG2

cells with or without YTHDF1 knockdown under high lipid stress. (d) FISH and IF showing that decreasing YTHDF1 expression reduced the sequestration of ACOX1 mRNA in SGs under high lipid stress. (e) Agarose gel electrophoresis analysis of the RIP-qPCR results showing that YTHDF1 depletion caused a significant decrease in the sequestration of ACOX1 mRNA in SGs. $n = 4$. (f) Immunofluorescence analysis of TIA-1 and G3BP1 to assess the effect of G3BP1 knockdown on SG formation. Knockdown of G3BP1 significantly reduced the number of SGs. (g) Western blot analysis and quantification of ACOX1 in G3BP1-knockdown HepG2 cells treated with PA and OA. $n = 3$. (h) Western blot analysis showing overexpression of YTHDF1-WT but not YTHDF1-K395A, Y397A decreased ACOX1 protein. The data are presented as the means ± SEMs. ns, no significance, $*p < 0.05$, $**p < 0.01$, $***p < 0.001$, and $****p < 0.0001$ (unpaired t test).

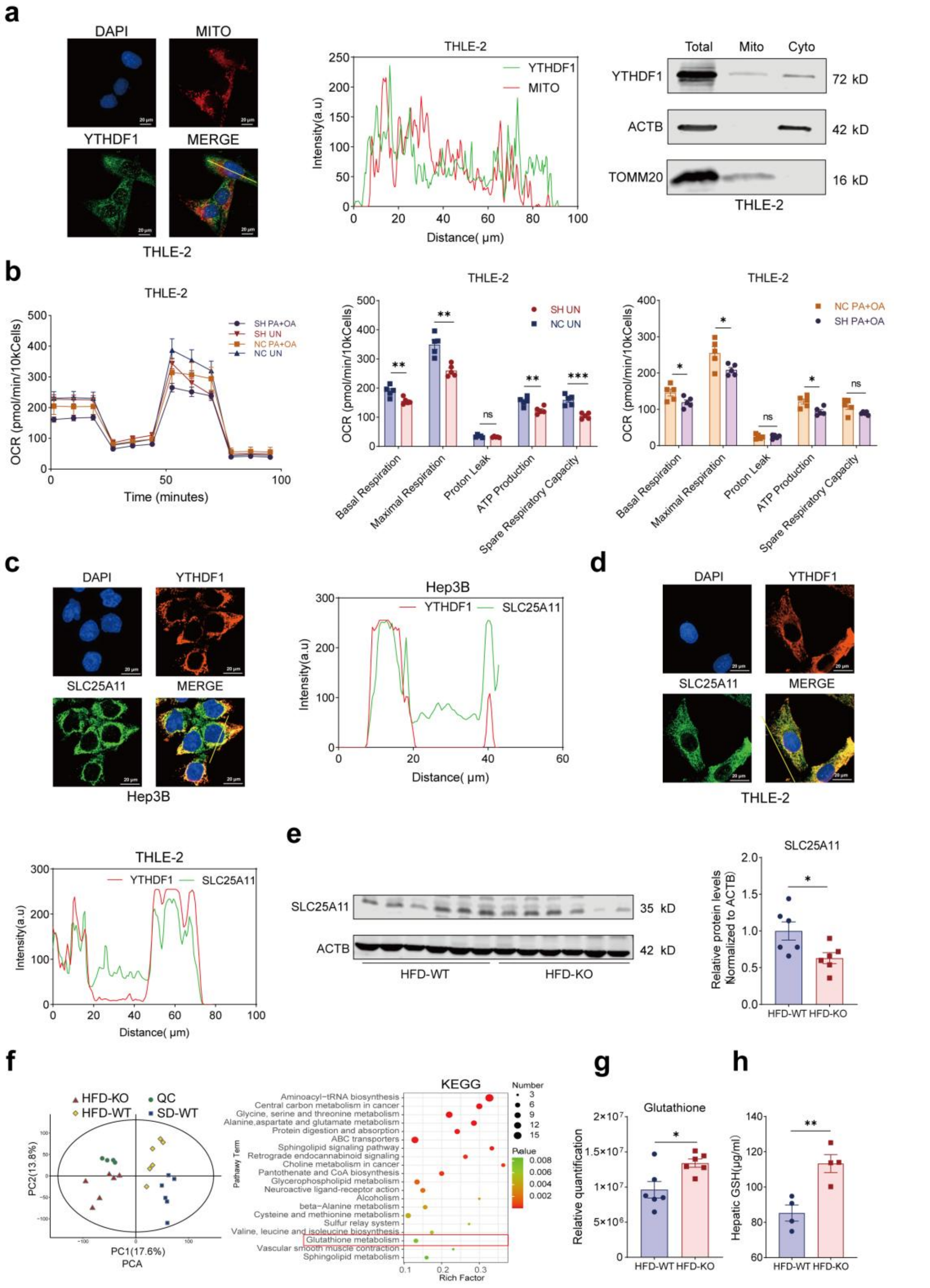

Supplementary Figure 7. **YTHDF1 depletion limits GSH delivery to mitochondria by decreasing SLC25A11 expression.** (a) IF staining and mitochondrial fractionation assays showing the localization of YTHDF1 in the mitochondria of THLE-2 cells. (b) The OCR in YTHDF1-silenced THLE-2 cells treated with PA and OA determined by

Seahorse. IF staining showing the colocalization between YTHDF1 and SLC25A11 in Hep 3B (c) and THLE-2 (d) cells. (e) Western blot analysis of SLC25A11 in hepatic tissues from HFD-fed WT and Ythdf1-KO mice. n=6. (f) Principal component analysis (PCA) and KEGG pathway analysis of metabolites in WT, HFD-fed WT, and HFD-fed Ythdf1-KO mice. (g) Quantitative results of glutathione in metabolomics data. (h) Specific quantitative assay kit for detecting glutathione content in liver tissues of HFD-fed WT and Ythdf1-KO mice. $n = 4$ or 6, respectively. The data are presented as the means ± SEMs. *$p < 0.05$, **$p < 0.01$ and ***$p < 0.001$ (unpaired t test).

Supplementary Table 3. The details of antibodies used in the study.

Antibody names	Brand	Product code
Anti-GAPDH	proteintech	10494-1-AP
Anti-ACOX1	ZEN BIO	R23371
Anti-YTHDF1	proteintech	17479-1-AP
Anti-ACCA1	proteintech	123919-2-AP
Anti-HSD17B4	proteintech	15116-1-AP
Anti-Mouse igG cy3	ZEN BIO	550075
Anti-Rabbit igG Alexa fluor 647	ZEN BIO	550048
Anti-G3BP1	proteintech	66486-1-lg
Anti-G3BP1	CST	61559
Anti-Mono Methyl lysine	ORIGENE	TA379599
Anti-PEX11a	proteintech	15481-1-AP
Anti-Catalase	proteintech	66765-1-lg
Anti-LAMP2	ZEN BIO	R381078
Anti-PLN2	proteintech	15294-1-AP
Anti-PMP70	abcam	Ab3421
Anti-SLC25A11	proteintech	12253-1-AP
Anti- mTOR	Cell signaling technology	mTOR Substrates Antibody Sampler Kit #9862
Anti- Phospho- mTOR	Cell signaling technology	mTOR Substrates Antibody Sampler Kit #9862
Anti-ULK1	Cell signaling technology	mTOR Substrates Antibody Sampler Kit #9862
Anti- Phospho-ULK1	ABcolonal	AP0736
Anti-β-actin	proteintech	66009-1-lg
IRdye680 Goat anti-mouse IgG	Licor	926-32220
IRdye800 Goat anti-rabbit IgG	Licor	926-32211
**Anti-LC3B**	Immunoway	YN552
Anti-p62	Immunoway	YM8025
Anti-PARK2/Parkin	proteintech	14060-1-AP
Anti-PINK1	proteintech	23274-1-AP

Supplementary Table 4. Sequences of primers for quantitative real-time PCR.

Primer	Sequences (5'- 3')
hum-YTHDF1-F	ACCTGTCCAGCTATTACCCG
hum-YTHDF1-R	TGGTGAGGTATGGAATCGGAG
hum-18S-F	GGAGAGGGAGCCTGAGAAACG
hum-18S-R	TTACAGGGCCTCGAAAGAGTCC
hum-ACTB-F	CATGTACGTTGCTATCCAGGC
hum-ACTB-R	CTCCTTAATGTCACGCACGAT
hum-ACOX1-F	GGAACTCACCTTCGAGGCTTG
hum- ACOX1-R	TTCCCCTTAGTGATGAGCTGG
mus-Acox1-F	TAACTTCCTCACTCGAAGCCA
mus-Acox1-R	AGTTCCATGACCCATCTCTGTC
mus-Acot1-F	ATACCCCCTGTGACTATCCTGA
mus-Acot1-R	CAAACACTCACTACCCAACTGT
mus-Acot2-F	GTTGTGCCAACAGGATTGGAA
mus-Acot2-R	GCTCAGCGTCGCATTTGTC
mus-Elovl2-F	CCTGCTCTCGATATGGCTGG
mus-Elovl2-R	AAGAAGTGTGATTGCGAGGTTAT
mus-Scd1-F	TTCTTGCGATACACTCTGGTGC
mus-Scd1-R	CGGGATTGAATGTTCTTGTCGT
mus-Abcd2-F	ATACACATGCTAAATGCAGCAGC
mus-Abcd2-R	GCCAATGATGGGATAGAGGGT
mus-Acaa1a -F	ACGCATCGCCCAATTTCTGA
mus-Acaa1a -R	CCAGACAGGGACATGGACTC
mus-Acad11-F	TGACACCGTGGAAGTGCTAC
mus-Acad11-R	CCCGGCAAGTGCTGATTCA
mus-Acot3-F	GTCGGGGTCCTTGGCATTT
mus-Acot3-R	GCCGATGTTGGATATAGAGCCAT
mus-Aldh3a2-F	CCTGAGCAAAAGTGAACTCAATG
mus-Aldh3a2-R	TCTTAGCCGGTCTCGCAGAA
mus-Crat-F	GCTGCCAGAACCGTGGTAAA
mus-Crat-R	CCTTGAGGTAATAGTCCAGGGA
mus-Decr2-F	CACGGCTGCTAAGAAGTTGGT
mus-Decr2-R	AGCTGCACAGTTAATGAGGATG
mus-Gstk1-F	GGTCCTATGCAGATACCAACAC
mus-Gstk1-R	GTACTGGCCTTTTCGGGGAA
mus-Hsd17b4-F	AGGGGACTTCAAGGGAATTGG
mus-Hsd17b4-R	GCCTGCTTCAACTGAATCGTAA
mus-Pex11a-F	AAGAGGCCGTGGTACTGAAG
mus-Pex11a-R	GGCTAATGTCAGGCATAAGCG
mus-Acaca-F	CTTCCTGACAAACGAGTCTGG
mus-Acaca-R	CTGCCGAAACATCTCTGGGA
mus-Srebf1-F	TGACCCGGCTATTCCGTGA
mus-Srebf1-R	CTGGGCTGAGCAATACAGTTC

mus-Fasn-F	GGAGGTGGTGATAGCCGGTAT
mus-Fasn-R	TGGGTAATCCATAGAGCCCAG
mus-Ppara-F	AGAGCCCCATCTGTCCTCTC
mus-Ppara-R	ACTGGTAGTCTGCAAAACCAAA
mus-Cpt1a-F	CTCCGCCTGAGCCATGAAG
mus-Cpt1a-R	CACCAGTGATGATGCCATTCT
mus-18s-F	GGAGAGGGAGCCTGAGAAACG
mus-18s-R	TTACAGGGCCTCGAAAGAGTCC
mus-Actb-F	GGCTGTATTCCCCTCCATCG
mus-Actb-R	CCAGTTGGTAACAATGCCATGT
